# Bacterial alkylquinolone signaling contributes to structuring microbial communities in the ocean

**DOI:** 10.1186/s40168-019-0711-9

**Published:** 2019-06-17

**Authors:** Kristen E. Whalen, Jamie W. Becker, Anna M. Schrecengost, Yongjie Gao, Nicole Giannetti, Elizabeth L. Harvey

**Affiliations:** 1 Department of Biology, Haverford College, Haverford, PA USA; 2 Skidaway Institute of Oceanography, University of Georgia, Savannah, GA USA

**Keywords:** Quorum sensing, 2-heptyl-4-quinolone, Microbiome, Phytoplankton, *Pseudoalteromonas*

## Abstract

**Background:**

Marine bacteria form complex relationships with eukaryotic hosts, from obligate symbioses to pathogenic interactions. These interactions can be tightly regulated by bioactive molecules, creating a complex system of chemical interactions through which these species chemically communicate thereby directly altering the host’s physiology and community composition. Quorum sensing (QS) signals were first described in a marine bacterium four decades ago, and since then, we have come to discover that QS mediates processes within the marine carbon cycle, affects the health of coral reef ecosystems, and shapes microbial diversity and bacteria-eukaryotic host relationships. Yet, only recently have alkylquinolone signals been recognized for their role in cell-to-cell communication and the orchestration of virulence in biomedically relevant pathogens. The alkylquinolone, 2-heptyl-4-quinolone (HHQ), was recently found to arrest cell growth without inducing cell mortality in selected phytoplankton species at nanomolar concentrations, suggesting QS molecules like HHQ can influence algal physiology, playing pivotal roles in structuring larger ecological frameworks.

**Results:**

To understand how natural communities of phytoplankton and bacteria respond to HHQ, field-based incubation experiments with ecologically relevant concentrations of HHQ were conducted over the course of a stimulated phytoplankton bloom. Bulk flow cytometry measurements indicated that, in general, exposure to HHQ caused nanoplankton and prokaryotic cell abundances to decrease. Amplicon sequencing revealed HHQ exposure altered the composition of particle-associated and free-living microbiota, favoring the relative expansion of both gamma- and alpha-proteobacteria, and a concurrent decrease in Bacteroidetes. Specifically, *Pseudoalteromonas* spp., known to produce HHQ, increased in relative abundance following HHQ exposure. A search of representative bacterial genomes from genera that increased in relative abundance when exposed to HHQ revealed that they all have the genetic potential to bind HHQ.

**Conclusions:**

This work demonstrates HHQ has the capacity to influence microbial community organization, suggesting alkylquinolones have functions beyond bacterial communication and are pivotal in driving microbial community structure and phytoplankton growth. Knowledge of how bacterial signals alter marine communities will serve to deepen our understanding of the impact these chemical interactions have on a global scale.

**Electronic supplementary material:**

The online version of this article (10.1186/s40168-019-0711-9) contains supplementary material, which is available to authorized users.

## Background

Interactions between phytoplankton and bacteria play a central role in mediating biogeochemical cycling and microbial trophic structure in the ocean. The associations between phytoplankton and bacteria are complex and can be both temporally variable [1] and species-specific [2]. These interactions can range from mutualistic, as bacteria and phytoplankton can support the growth of one another via the exchange or recycling of nutrients [3], to pathogenic—resulting in decreased chlorophyll biosynthesis and photosynthesis, induction of caspase-like activity, cell wall lysis [4], and ultimately phytoplankton mortality [5]. Even within a single bacterial-algal interaction, both modalities can be observed as bacteria sense and respond to their eukaryotic hosts [6]. Given that both bacteria and phytoplankton grow exponentially, even slight decreases in growth rate have the potential to dramatically influence population dynamics, ultimately influencing large-scale oceanic processes, including phytoplankton bloom dynamics.

There is increasing recognition that exuded small molecules can mediate marine microbial interactions through a variety of mechanisms, including directing communication, influencing mate-finding, inducing defenses, modifying behavior, and causing mortality [4, 7–11]. Originally described in a marine bacterium four decades ago [12], quorum sensing (QS) is a form of chemical communication by which bacteria can, in unison, coordinate gene expression and induce density-dependent cooperative behavior. This behavior is triggered by small, diffusible chemical signals secreted by bacteria that initiate group-beneficial behaviors after accumulating to appreciable threshold levels [13]. Our knowledge of the battery of genes that can be induced by QS signals originates from understanding their roles in human pathogenic bacteria, whereby QS-mediated pathogenicity of Gram-negative bacteria is attributed to the production and secretion of virulence factors (i.e., tissue degrading enzymes, endotoxins, exotoxins, siderophores, adherence components) [14]. Additional behaviors coordinated by QS molecules aid in colonization, nutrient acquisition, and collective defenses, including changes in bacterial motility that may allow bacteria to access resources, form biofilms at densities up to three orders of magnitude greater than planktonic bacteria, and produce exopolysaccharides for adhesion, extracellular, hydrolytic enzymes, and antibiotics [13, 15].

In Gram-negative bacteria, acyl-homoserine lactone (AHL)-mediated quorum signaling is the best understood QS signaling system, which consists of a homoserine lactone with a fatty acid side chain. However, 20 years ago, a second alkylquinolone-based QS signaling system was described in *Pseudomonas aeruginosa* as part of this signaling system, and the antibiotic alkylquinolone, 2-heptyl-4-quinolone (HHQ), was discovered [16]. Since the discovery of HHQ and the key role this QS molecule has in coordinating virulence via activation of canonical transcriptional regulators, additional studies have demonstrated HHQ can repress both motility and biofilm formation in bacteria and yeast, and exhibit potent bacteriostatic activity against several Gram-negative bacteria, including pathogenic *Vibrio vulnificus* [17]. This work revealed HHQ acts as a novel interkingdom signal, having both the ability to coordinate molecular circuitry and cellular function in *P. aeruginosa*, as well as function in an antagonistic fashion towards other microorganisms, indicating a role for HHQ in mediating polymicrobial communities from diverse environmental niches [17, 18].

Recently, marine bacteria within the genera *Pseudoalteromonas* and *Pseudomonas* were shown to produce HHQ [19]. Additionally, the finding that nanomolar concentrations of HHQ arrests cell growth without inducing cell mortality in phytoplankton in a species-specific manner [19] suggests alkylquinolones have a more widespread influence on microbial and eukaryotic systems than previously appreciated. Similar to AHLs, which are known to be involved in bacterial cross-talk and influence eukaryotic development, HHQ appears to have an impact on microbial-eukaryotic host interactions; however, the molecular underpinnings of these interactions are yet unknown. Studies of human pathogenic bacteria have provided the fundamental knowledge needed for understanding how alkylquinolones engage bacteria in cooperative and coordinated behaviors [17, 18, 20–22], yet parallel studies in marine systems investigating how alkylquinolones influence marine microbial population dynamics are still emerging.

To date, studies of HHQ in relation to the marine environment have focused on laboratory experiments involving specific phytoplankton-bacteria interactions and cannot provide a comprehensive view of the community-wide response of natural microbes to this signaling molecule. In order to develop an understanding of how the alkylquinolone HHQ influences growth rates and the community composition of marine microbes in situ, we conducted field-based incubation experiments where natural assemblages were exposed to ecologically relevant concentrations of HHQ.

## Results and discussion

**Fig. 1:**
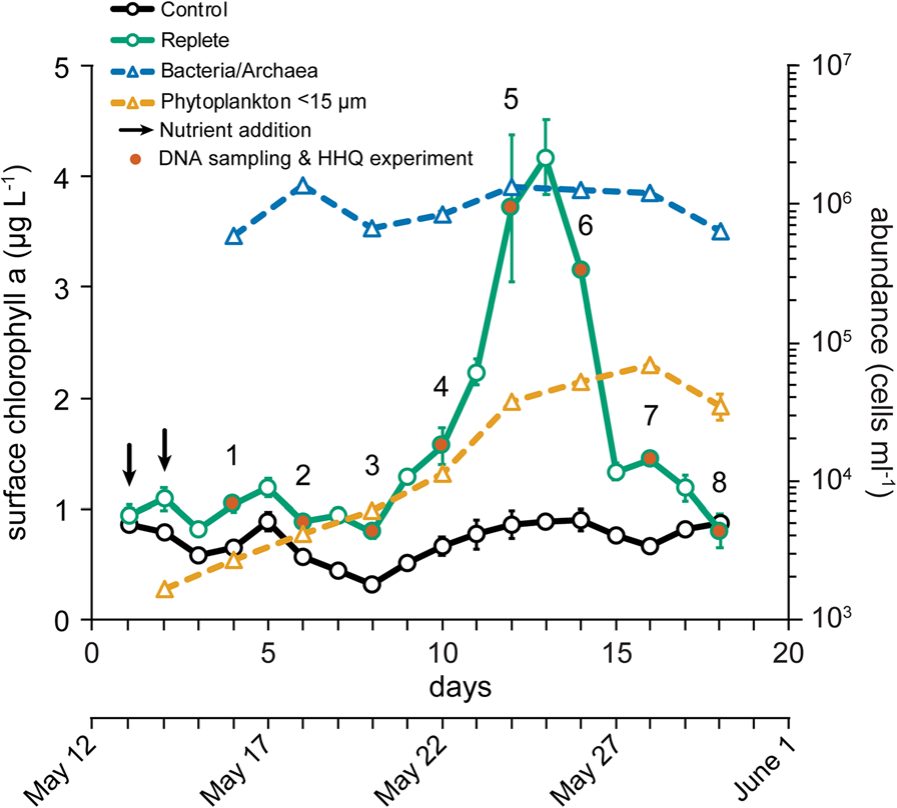
Mesocosm chlorophyll *a* and microbial cell concentrations. Chlorophyll *a* samples were taken from nutrient replete (green circles and black arrows) and unamended control mesocosms (black circles). Samples for total bacteria/archaea (blue triangles) and total phytoplankton < 15 μm (orange triangles) cell abundances were taken from replete mesocosms. DNA sampling and 2-heptyl-4-quinolone manipulations occurred at eight experimental time points (labeled 1 through 8) during sampling (red circles). All samples were taken from a depth of 1 m and symbols represent the mean (± s.d.) of biological triplicates.

Our field-based study was designed to describe (i) how bacterial/archaea and phytoplankton populations might be perturbed in the presence of the bacterial signaling molecule, HHQ, and (ii) how variable this response is to HHQ by examining community-level changes over the course of a coastal phytoplankton bloom. Our efforts to stimulate a phytoplankton bloom were successful, as evidenced by elevated surface chlorophyll *a* concentrations in replete mesocosms relative to unamended control mesocosm bags (Fig. 1). At the peak of the bloom, picoeukaryotes were the most numerically abundant group observed (Additional file 1: Figure S1). Bulk free-living bacteria/archaea abundance measurements remained relatively constant over the course of the bloom (Fig. 1). Similar trends in pico-, nano-, and bacterio-plankton abundances following nutrient additions have been observed prior in this same ecosystem [23]. Water from three nutrient replete mesocosm bags was pooled for DNA sampling at eight time points distributed over the course of the bloom to investigate prokaryotic (free-living and particle-associated) and eukaryotic phytoplankton community dynamics during the bloom and after exposure to an environmentally relevant concentration of HHQ (Fig. 1).

### Bloom dynamics influence microbial diversity and abundance

**Fig. 2:**
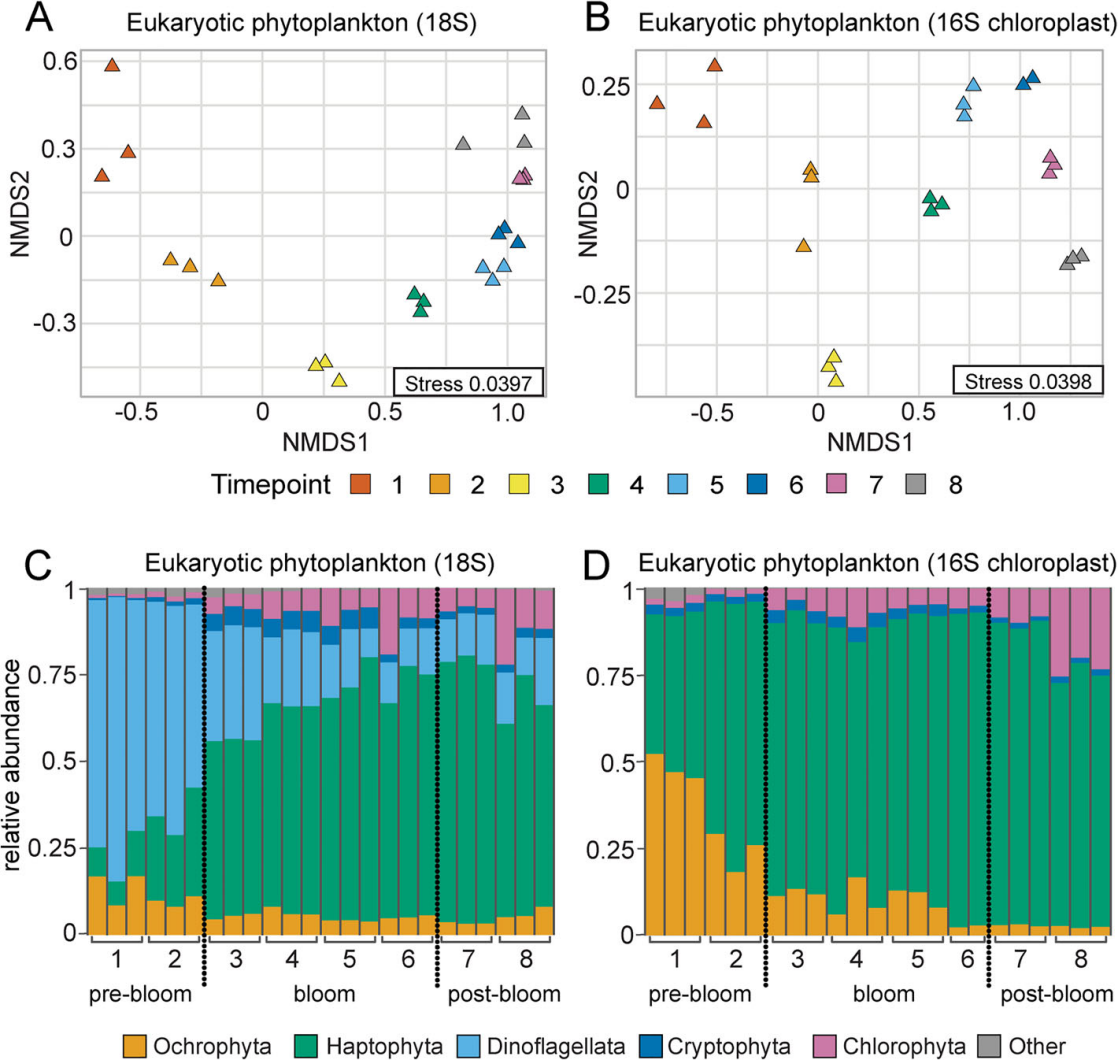
Nonmetric multidimensional scaling ordination displaying eukaryotic communities over the course of the bloom. Ordination of eukaryotic phytoplankton communities based on 18S rRNA (**a**) and 16S chloroplast (**b**) amplicon sequence variants visualized by non-metric multidimensional scaling (NMDS) of Bray-Curtis distance. Triangle color indicates experimental time points. Relative abundances of eukaryotic phytoplankton divisions (≥ 1%) determined by 18S rRNA (**c**) and 16S chloroplast (**d**) amplicon sequence variants. X-axes indicate experimental time points.

Non-metric multidimensional scaling (NMDS) analysis of 18S rRNA and 16S chloroplast amplicon sequences based on Bray-Curtis dissimilarity between samples indicated clear sample clustering by time point, in which the date of sampling during the bloom explained 92% and 95% of the variability, respectively (permutational multivariate analysis of variance (PERMANOVA), *p* = 0.001 for both data sets; Fig. 2a, b). Over the course of the bloom, there was a transition from a dinoflagellate-dominated community, specifically *Blecheleria* sp., to a haptophyte (*Chrysochromulina* sp., *Prymnesium* sp.)- and chlorophyte (*Micromonas* sp.)-dominated community (Fig. 2c, d). Since estimates of eukaryotic relative abundances can be confounded by taxonomic variations in 18S rRNA copy number, we also examined 16S plastid amplicon sequence variants (ASVs) and found a significant (*p* < 0.001) positive correlation (Spearman’s correlation coefficient = 0.66–0.76) with the 18S data among the dominant eukaryotic phytoplankton divisions after removal of sequences assigned to the Dinoflagellata division (Additional file 2: Figure S2). The discontinuity between the 18S rRNA and 16S plastid sequence results with respect to the Dinoflagellata results from the fact that most of the chloroplast genes have been lost to the nucleus in dinoflagellate algae, while still retaining a cytologically recognizable chloroplast [24]. Moreover, during the course of the bloom, cyanobacterial abundance increased with *Synechococcus* sp. dominating during and after the peak of the bloom (Additional file 3: Figure S3).

**Fig. 3:**
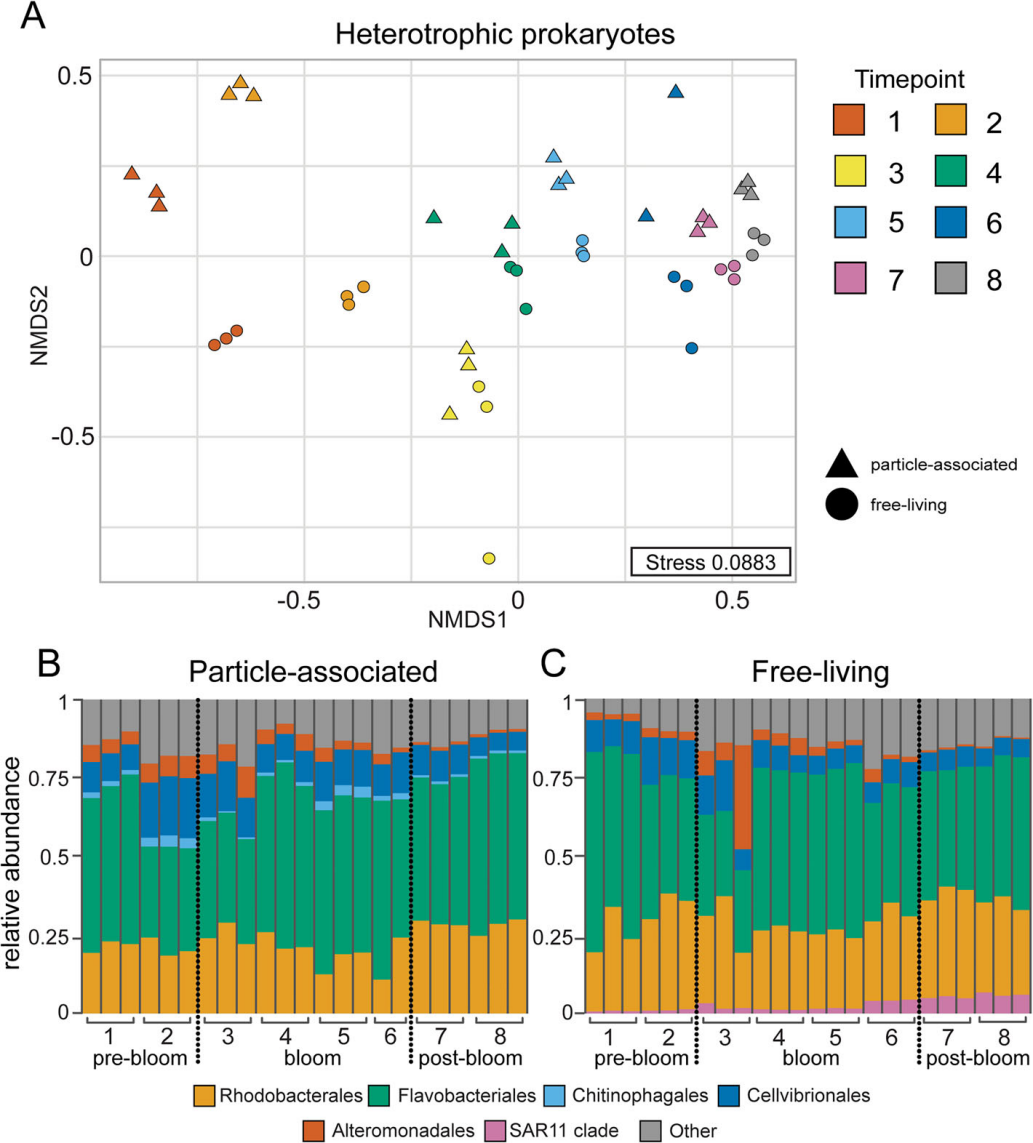
Nonmetric multidimensional scaling ordination displaying microbial communities over the course of the bloom. Ordination of heterotrophic prokaryote communities based on 16S rRNA amplicon sequence variants (**a**) visualized by non-metric multidimensional scaling (NMDS) of Bray-Curtis distance. Triangles and circles correspond to particle-associated (> 1 μm size) and free-living communities, respectively. Symbol color denotes experimental time point. Relative abundances of heterotrophic prokaryote orders (≥ 1%) determined by 16S rRNA amplicon sequence variants for particle-associated (> 1 μm size) (**b**) and free-living (**c**) communities. X-axes indicate experimental time points.

Analysis of particle-associated and free-living prokaryotes based on 16S rRNA amplicon sequencing indicated that, similar to eukaryotic phytoplankton communities, the majority (75%) of the variability was explained by the date of sampling during the bloom (PERMANOVA, *p* = 0.001; Fig. 3a). Size-fractionation explained 9% of the variation (PERMANOVA, *p* = 0.008), with the greatest separation between particle-associated and free-living prokaryotic communities observed prior to the initiation of the bloom (i.e., experiments 1 and 2; Fig. 3a). Automated FlowCam analysis of mesocosm water samples revealed increased numbers of cellular aggregates over the bloom phase (i.e., experiments 3–6). These cellular aggregates continued to be observed in the post-bloom phase with an increased amount of phytoplankton debris and fecal pellets (Additional file 1: Figure S1a). Our data suggest that homogenization of particle-associated and free-living prokaryotic communities during and especially after the bloom may be a result of a distinct shift in the eukaryotic phytoplankton community that serve as prokaryotic reservoirs for specific species, and/or the subsequent increase in particulate organic material that was generated in response to bloom conditions that may also serve as surfaces for transient free-living representatives to colonize [25]. The most abundant orders of bacteria present in both particle-associated and free-living samples were the *Rhodobacterales* and *Flavobacteriales*, with the relative abundances of these major groups oscillating over the course of the bloom (Fig. 3b, c). Representatives from the SAR11 clade generally increased in abundance in the free-living fraction over the course of the bloom, similar to previous reports [26], while the orders *Chitinophagales*, *Cellvibrionales*, and *Alteromonadales* all decreased in relative abundance in the particle-associated fraction over the bloom.

### HHQ impacts on microbial community structure

**Fig. 4:**
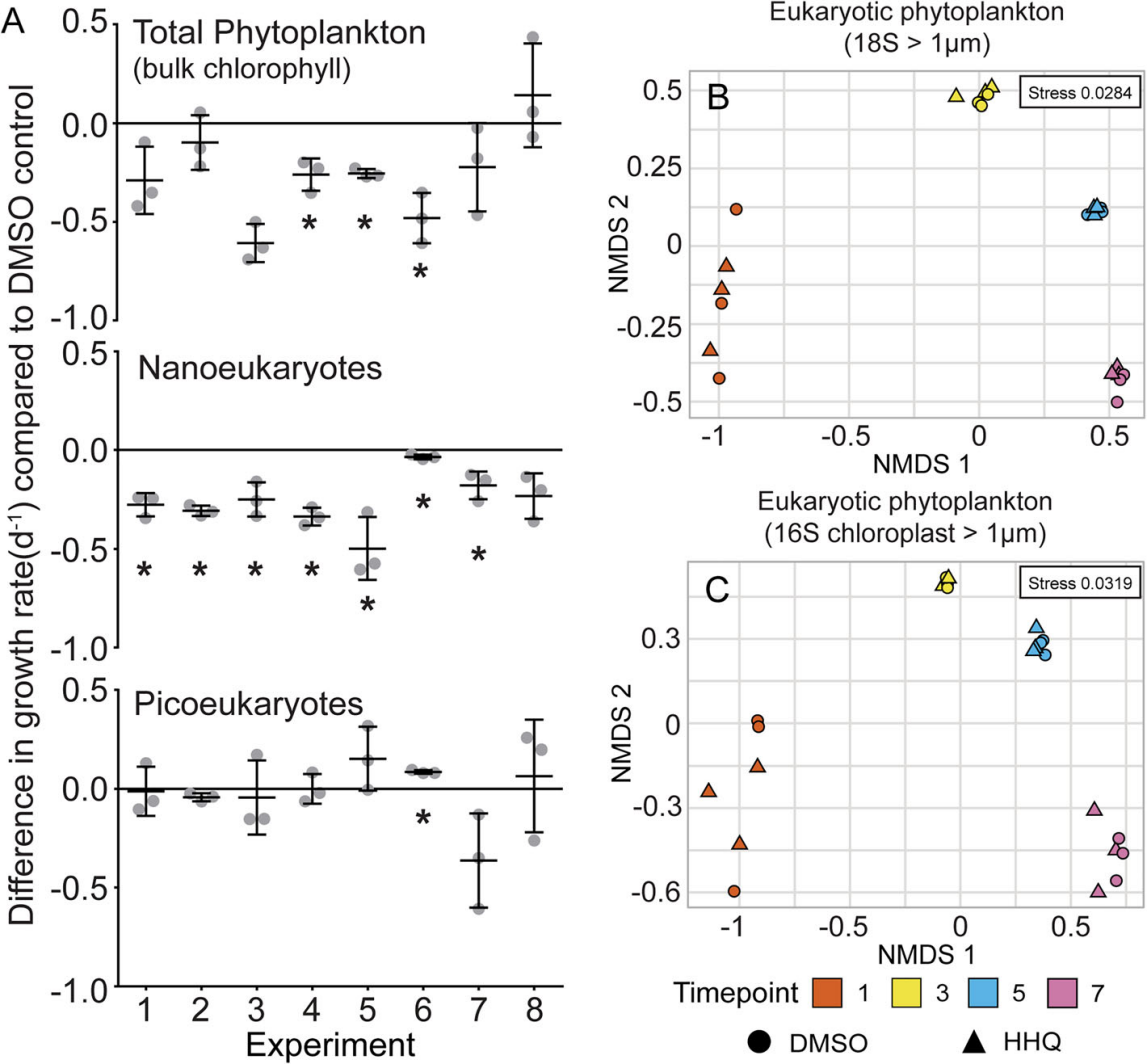
Response of phytoplankton communities to HHQ exposure. Difference in growth rate (d^−1^) in phytoplankton abundance after 24 h exposure to 410 μM (100 ng mL^−1^) 2-heptyl-4-quinolone (HHQ) compared to the DMSO control determined by chlorophyll a concentration, or flow cytometry counts of nano- and pico-eukaryotes (**a**) over eight experimental time points. Bars represent the mean (± s.d.) of biological triplicates and asterisks indicate significant changes in phytoplankton growth rate between the two treatments (*p* < 0.05). Ordination of eukaryotic phytoplankton communities based on 18S rRNA (**b**) and 16S chloroplast (**c**) amplicon sequence variants visualized by non-metric multidimensional scaling (NMDS) of Bray-Curtis distance. Circles and triangles correspond to DMSO control and HHQ exposed communities, respectively. Symbol color denotes experimental time point.

When mesocosm communities were exposed to nanomolar concentrations of HHQ (410 nM or 100 ng/mL) for 24 h, significant (*p* < 0.05) reductions in population-level growth rates of eukaryotic phytoplankton, relative to a DMSO control, were observed (Fig. 4a). Specifically, HHQ exposure caused the growth rate of phytoplankton—based on changes in bulk chlorophyll *a* concentration—to decrease significantly over the peak of the bloom only, whereas the growth rate of HHQ exposed nanoeukaryotes was significantly lower than the control at all time points except experiment 8. Picoeukaryotes did not exhibit a consistent, significant pattern in growth rate response to HHQ exposure.

These results suggest species-specific growth responses to HHQ, which have been observed in previous laboratory experiments [19]. Interestingly, there was only one significant response of picoeukaryotes to HHQ (experiment 6), yet they comprised the highest percentage of the total phytoplankton in terms of cell density and carbon biomass during the peak and post-bloom. Conversely, while nanoeukaryotes increased in population abundance over the course of the bloom, the percent contribution of this group to total cell counts and carbon biomass decreased. Due to their small size and higher growth rates, picoeukaryotes often have a competitive advantage over other co-occurring groups of phytoplankton [27, 28]. The addition of nitrate and phosphate at the beginning of the mesocosm experiment could have promoted picoeukaryote growth over nanoeukaryotes. It is impossible to detangle if HHQ was more impactful to nanoeukaryotes because this group has enhanced susceptibility, or if fast-growing populations, such as the picoeukaryotes, are not impacted by HHQ. Thus, the impact of HHQ on natural populations may be mediated by cell physiological parameters as well as community composition.

NMDS analysis of 18S rRNA, 16S chloroplast, and 16S cyanobacterial amplicon sequences indicated clear clustering of samples that were exposed to HHQ with those taken from the DMSO control at the same time point, indicating that HHQ exposure had a minimal impact on the community composition of eukaryotic phytoplankton and cyanobacteria (PERMANOVA, *p* > 0.1; Fig 4b, c, Additional file 2: Figure S2). Additionally, no significant differences in the relative abundance of any eukaryotic ASVs were observed following HHQ exposure at any experimental time point. This data in combination with the earlier finding articulating how HHQ resulted in a significant decrease in both bulk chlorophyll and nanoeukaryotic growth rates suggests that HHQ reduced eukaryotic cell growth across all nanoeukaryotes species but did not significantly change community composition. This finding is consistent with earlier work demonstrating HHQ causes the arrest of cellular growth rather than lysing phytoplankton cells [19], which would be lost from the system. Further, given the short duration of these experiments, it is possible that phytoplankton communities exposed to HHQ for longer periods of time may experience distinct shifts in community composition, as those groups not influenced by HHQ would opportunistically outcompete those cemented in arrested growth.

**Fig. 5:**
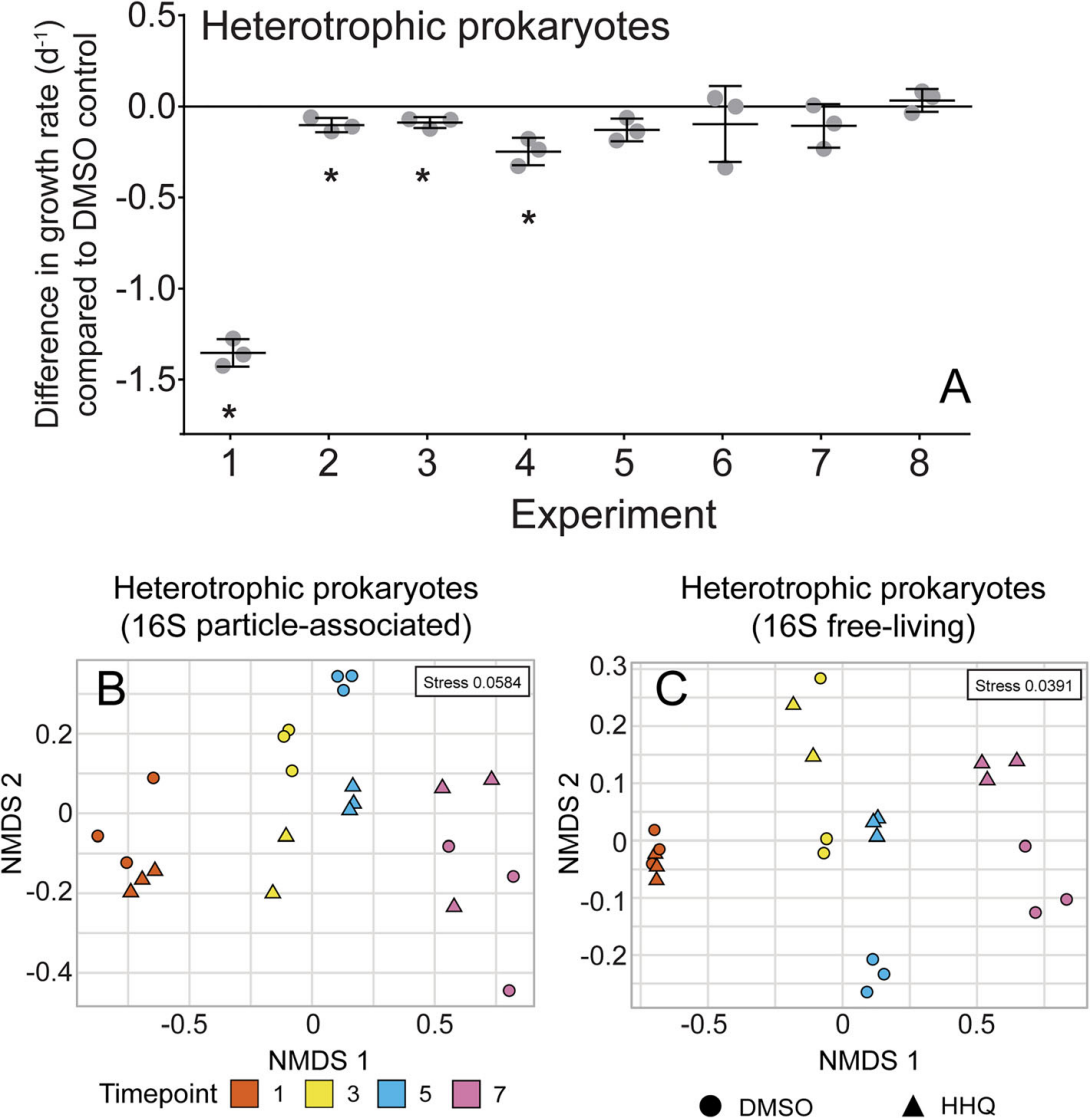
Response of microbial communities to HHQ exposure. Difference in growth rate (d^−1^) in heterotrophic prokaryote abundance after 24 h exposure to 410 μM (100 ng mL^−1^) 2-heptyl-4-quinolone (HHQ) compared to the DMSO control determined by flow cytometry over eight experimental time points (**a**). Bars represent the mean (± s. d.) of biological triplicate and asterisks indicate significant changes in the growth rate of the bulk communities between the two treatments (*p* < 0.05). Ordination of heterotrophic prokaryote communities based on particle-associated 16S rRNA (**b**) and free-living 16S rRNA (**c**) amplicon sequence variants visualized by non-metric multidimensional scaling (NMDS) of Bray-Curtis distance. Circles and triangles correspond to DMSO control and HHQ exposed communities, respectively. Symbol color denotes experimental time point.

For bacteria/archaea, the addition of HHQ resulted in changes in growth rate and diversity. When exposed to HHQ, the growth rate of the bulk microbial population was significantly (*p* < 0.05) slower than the DMSO control at the beginning of the bloom (experiments 1–4; Fig. 5a). NMDS analysis of 16S rRNA amplicon sequences indicated potential separation between the DMSO control and HHQ addition treatments during the peak of the bloom (Fig. 5b, c). While HHQ was not a significant driver of the overall observed variation (PERMANOVA, *p* > 0.1), removal of pre- and post-bloom samples (experiments 1 and 7) from the ordinations revealed that the HHQ treatment explained 31% (*p* = 0.013) and 25% (*p* = 0.053) of the variation among the remaining particle-associated and free-living heterotrophic prokaryotic communities, respectively. Overall, these patterns indicate that HHQ impacted the growth and subsequent diversity of both particle-associated and free-living bacteria/archaea communities in the early and peak phases of the bloom, highlighting the role of HHQ as a potential driver of microbial diversity under bloom conditions.

**Fig. 6:**
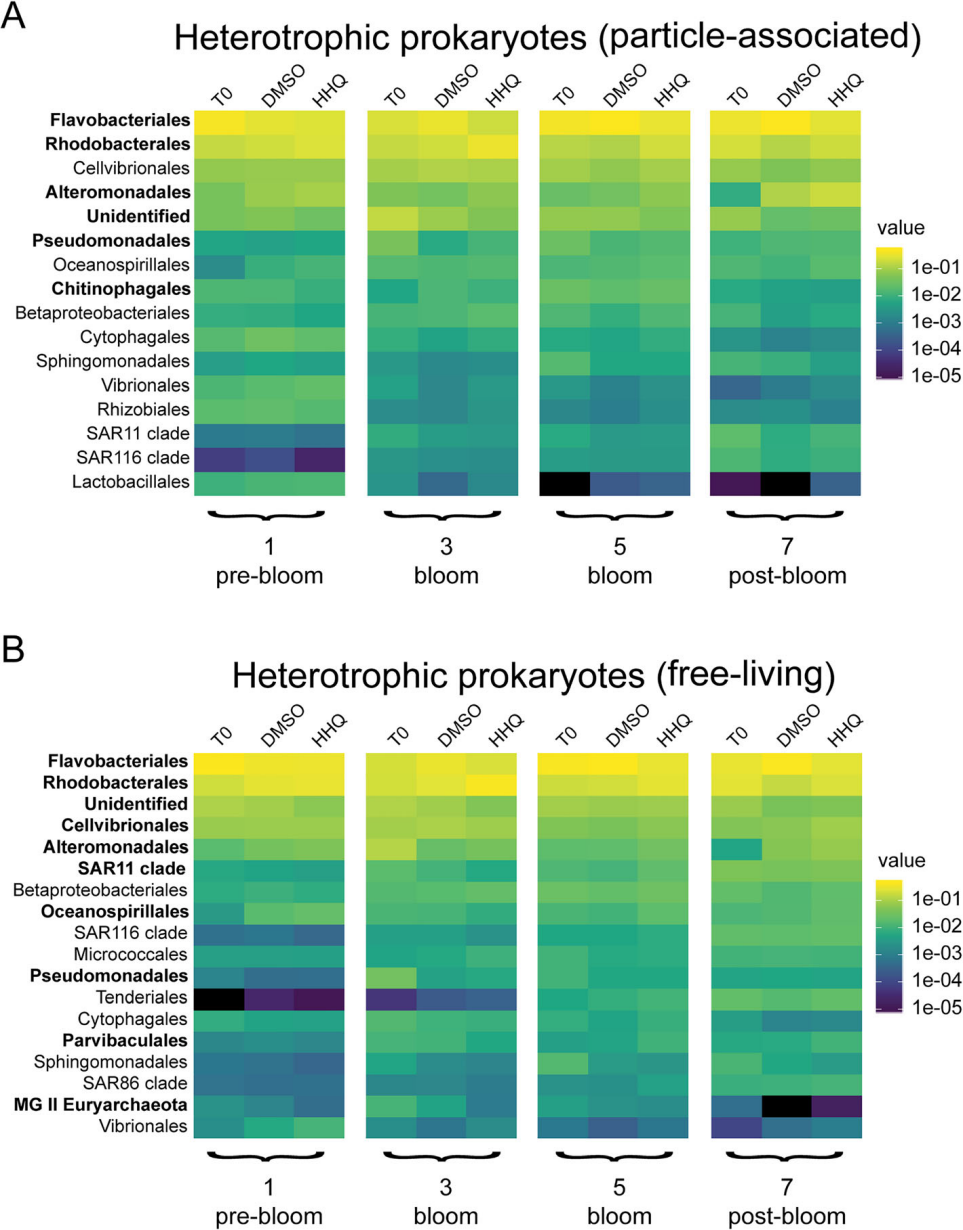
Heatmap showing average relative abundances of microbial communities after HHQ exposure. Relative abundance of heterotrophic prokaryotes in particle-associated (> 1 μm) (**a**) and free-living (**b**) communities for orders representing ≥ 1% of the community in at least one sample. Experimental time points and bloom phase is noted below each heatmap. Each column represents the mean of triplicate samples taken from replete mesocosms (*T*_0_) or exposed for 24 h to DMSO or 2-hepyl-4-quinolone (HHQ). Bolded taxa contain amplicon sequence variants that changed (+/−) significantly (BH-adjusted *p* value < 0.1) in relative abundance after HHQ exposure compared to the DMSO control.

**Fig. 7:**
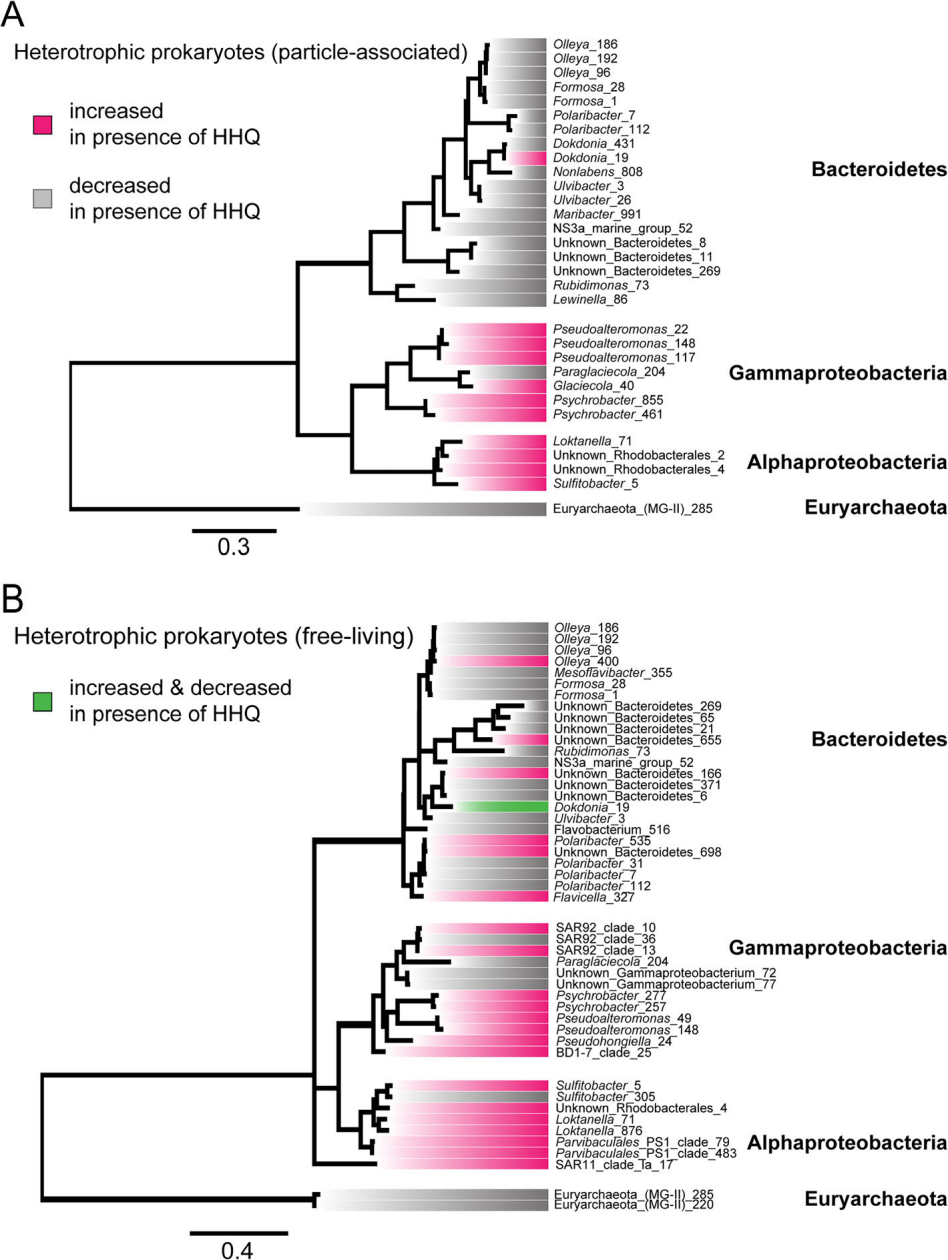
Amplicon sequence variants showing significant changes in their relative abundance following HHQ exposure. Phylogenetic relationship of amplicon sequence variants (ASVs) that significantly (BH-adjusted *p* value < 0.1) increased (magenta) or decreased (gray) in relative abundance after 24 h exposure to 2-heptyl-4-quinolone (HHQ). One ASV showing both an increase and decrease at different time points is labeled in green. Sequences were aligned using MAFFT and maximum-likelihood phylogenetic inference was done using RAxML.

Interestingly, *Flavobacteriales* representatives in both particle-associated and free-living fractions generally decreased in their relative abundance upon exposure to HHQ during and after the bloom, in contrast to representatives of the *Rhodobacterales* and *Alteromonadales* orders, which significantly increased in relative abundance (BH-adjusted *p* value < 0.1) in response to HHQ in both size fractions (Figs. 6 and 7; Additional file 3: Figure S3 and Additional file 4: Figure S4; Additional file 9: Table S1). It is important to note that the changes in relative abundance we report should not be interpreted as changes in absolute abundance due to the inherent compositional nature of the underlying data. Other orders with ASVs that significantly increased in relative abundance upon HHQ exposure in free-living fractions included *Cellvibrionales*, *Nitrosococcales*, *Parvibaculales*, SAR11, *Oceanospirillales*, *Betaproteobacteriales*, and *Tenderiales* (Additional file 9: Table S1). Representatives from the order *Pseudomonadales* were significantly higher in relative abundance in both the free-living fraction in post-bloom samples and particle-associated fraction in pre-bloom samples. In contrast, groups showing significantly lower relative abundances due to HHQ exposure included representatives from the Marine Group (MG) II *Euryarchaeota*, *Chitinophagales*, and additional ASVs that could not be identified at the order level (Additional file 9: Table S1). *Rhodobacterales*, *Flavobacteriales*, *Cellvibrionales*, and *Alteromonadales* orders all contained individual ASVs that responded in opposite directions when exposed to HHQ; however, only one ASV in the *Flavobacteriaceae* family (*Dokdonia_*ASV_19) significantly responded both negatively and positively to HHQ depending on the phase of the bloom (Fig. 7). The complexity of these signals, in which an ASV changes its response depending on the bloom phase and distinct ASVs within a single genus display disparate trends, highlights the advantage of using ASVs as opposed to binning similar amplicons into operational taxonomic units (OTUs), as these signals would be lost at broader taxonomic resolutions.

### Searching for alkylquinolone binding partners in HHQ-responsive bacteria

While diffusible signals coordinate cellular activities to the benefit of the producing organism, often competitors are “listening in,” decoding, and disarming these messages in heterogeneous microbial communities [17]. This concept of interspecies signaling has evolved from the biomedical literature with respect to how signaling molecules influence the outcome of infectious diseases [18]; however, parallels can be drawn to marine host-microbe communities as well. Of the 55 alkylquinolones known to be produced via the PqsABCDE biosynthetic pathway in *P. aeruginosa* [18], we are beginning to understand their functions beyond their QS capacity, including their ability to act synergistically to inhibit bacterial growth [29], iron chelation, and antimicrobial activity [18]. Alkylquinolones, like HHQ, facilitate the emergence of *P. aeruginosa* in complex biofilms but have also been found to modulate the virulence behavior of different pathogens, or inhibit biofilm formation in Gram-positive bacteria and serve as a bacteriostatic agent to several Gram-negative species including *Vibrio* spp. [18]. Moreover, co-colonizing microbes have developed the ability to degrade HHQ or quench alkylquinolone signals, indicating the influence of these molecules in the polymicrobial community [18, 22]. Our results in light of these findings indicate that HHQ could interact directly with non-producing alkylquinolone bacterial species and serves to highlight the importance of alkylquinolone chemical messages in structuring microbial communities by both promoting and hindering microbial growth. When examining general trends between particle-associated and free-living bacteria displaying significant changes in response to HHQ, *Proteobacteria* in the gamma and alpha classes (i.e., *Psychrobacter*, *Sulfitobacter*, *Glaciecola*) were more apt to increase their abundance in response to HHQ, suggesting these genera may have the capacity to respond to alkylquinolone signals (Additional file 4: Figure S4, Additional file 5: Figure S5, Additional file 6: Figure S6). Genomes from bacterial representatives closely related (98.6–99.4% partial 16S rRNA gene sequence identity) to those ASVs found to significantly increase (BH-adjusted *p* value < 0.1) in response to HHQ were mined for the LysR family regulator PqsR (also known as MvfR) from *Pseudoalteromonas piscicida* (strain A757, GenBank Acc. No. KT879198). PqsR has been shown to be activated by HHQ and is responsible for controlling ~ 140 genes, including several virulence factors (i.e., pyocyanin, LecA, and elastase) in *P. aeruginosa* [22]. This search revealed representatives from the genera *Psychrobacter*, *Sulfitobacter*, and *Glaciecola*, all contain putative LysR-type family regulators with homology to the HHQ-responsive PqsR transcriptional regulator (Additional file 10: Table S2), indicating the potential of these species to respond to environmental chemical signals.

Previously, a proteomic level screen in *P. aeruginosa* was used to identify binding partners for alkylquinolones [14], including HHQ, and was used in this study to identify molecular targets of interspecies signaling. This study identified three additional targets (WbpB, FtsZ, and AstB) that are under QS control and are potential binders of HHQ [14]. Searches for homologous sequences in the genomes of bacteria related to the ASVs that increased in relative abundance following HHQ exposure found homologs for WbpB, FtsZ, and AstB in *Psychrobacter*, *Sulfitobacter*, and *Glaciecola* strains (Additional file 10: Table S2). WbpB and FtsZ are suggested to be an “off target” binding partner of alkylquinolones. Previous work has shown alkylquinolones can affect colony morphology [14], and FtsZ function has been linked to alteration in colony phenotypes, as FtsZ is a tubulin-like protein that forms part of the septum in the contractile ring during cell division [30]. WbpB is thought to be involved in synthesizing sugar derivatives important in protein glycosylation involved in lipopolysaccharide biosynthesis and may interface in pathways that govern virulence factor production [31], and AstB is the second enzyme of the arginine succinyltransferase pathway involved in arginine catabolism for nitrogen utilization [32]. While further work is needed to explore the impact of HHQ on those genera shown to be responsive in our field studies, mining of genomic data from bacterial representatives has led to the identification of several promising putative HHQ targets homologs warranting further investigation.

There is increasing evidence that cross-communication involving QS signaling molecules exists in complex marine microbial communities in biofilms, and similarly, may likely contribute to structuring the phycosphere. We now recognize that organisms can degrade or quench QS signals by blocking steps in the signaling pathway, which has been extensively described for AHLs [33]. Recently, the supernatant of *Psychrobacter* sp. (B98C22) isolated from a marine sponge was able to inhibit AHL activity and impair the formation and stability of *P. aeruginosa* PA14 and *Bacillus subtilis* biofilms, suggesting this genus has the ability to quench QS signaling [34]. In addition, the marine bacterial species *Sulfitobacter* (family *Rhodobacteraceae*) and *Glaciecola* (family *Alteromonadaceae*) both isolated from *Ulva* colonized rocks were found to produce AHLs [35]. However, when *Sulfitobacter* (isolate 5) was grown in the presence of an AHL-degrading bacterium, its AHL production was severely disrupted [35]. These findings suggest *Psychrobacter*, *Sulfitobacter*, and *Glaciecola* are either able to quench QS signals or associate with species that do and in light of additional examples [36] of QS interference/quenching offer insight into the complexity of chemical signaling in heterogeneous microbial communities on surfaces. Moreover, enzymes involved in alkylquinolone degradation have recently been identified [22], emphasizing the idea that sympatric bacteria can disarm a possible alkylquinolone threat [18]. Future efforts aimed at understanding the crosstalk between QS signaling systems in free-living marine microbial communities and polymicrobial biofilms associated with phytoplankton hosts, in terms of who is producing signaling molecules and those capable of listening for them, will help to elucidate the underlying mechanisms that dictate microbial community structures. Our results reveal several marine bacterial genera that appear responsive to HHQ signaling for further investigation into HHQ-mediated crosstalk.

Following HHQ exposure, representatives from the *Bacteroidetes* phylum (*Formosa*, *Olleya*, *Ulvibacter*) generally decreased in relative abundance (Additional file 6: Figure S6). These trends in the reduction of representative *Bacteroidetes* in response to HHQ were more consistent for particle-associated bacteria than free-living bacteria (Fig. 7), suggesting HHQ may influence surface-associated and free-living species differently. It is known from clinical studies that HHQ can negatively impact the growth of non-HHQ producing bacteria within a polymicrobial population [18]. However, as stated above, due to the compositional nature of our data, we are unable to determine if the reduction in relative abundance of these genera is due directly to HHQ exposure from coercive growth inhibition, or the increased relative abundance of HHQ-responsive bacteria resulting in these observed decreases, independent of any changes to their absolute abundances. Indeed, a third possibility exists in which the modulation of virulence or other key phenotypes in other opportunistic bacteria within the phycosphere could also cause the reduction in the relative abundance of these genera. Further experiments utilizing representative laboratory isolates from these genera are needed to disentangle these possible explanations.

### Response of *Pseudoalteromonas* spp. to HHQ exposure

**Fig. 8:**
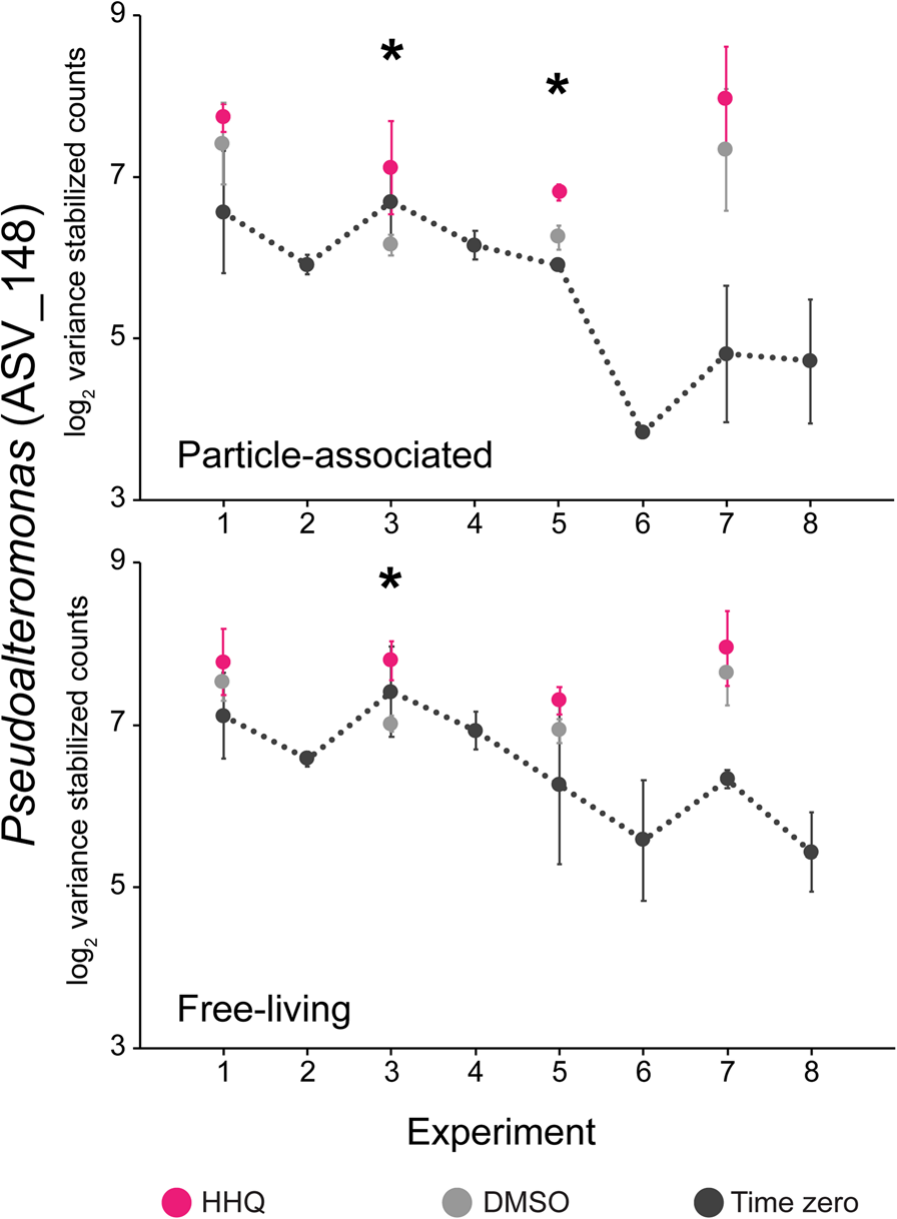
A representative *Pseudoalteromonas* ASV showing increased relative abundance following HHQ exposure. Normalized counts of *Pseudoalteromonas* ASV_148 from replete mesocosms at time zero (*T*_0_; black circles), and after 24 h exposure to either a solvent control (DMSO; gray circles) or 2-heptyl-4-quinolone (HHQ; magenta circles). Asterisks indicate significant differences in the relative abundance after HHQ exposure compared to the DMSO control (BH-adjusted *p* value < 0.1). X-axes indicate experimental time points.

Given that HHQ is produced by marine *Pseudoalteromonas* spp. [19, 37], we were interested in examining if naturally occurring marine *Pseudoalteromonas* spp. were capable of responding to HHQ. A key finding of the amplicon analysis revealed that *Pseudoalteromonas* ASVs significantly increased in relative abundance (BH-adjusted *p* value < 0.1) in both particle-associated and free-living fractions following HHQ exposure in comparison to DMSO controls (Fig. 7, Additional file 4: Figure S4, Additional file 5: Figure S5). For the particle-associated microbial fractions, *Pseudoalteromonas* ASVs significantly increased in relative abundance after HHQ exposure at time points corresponding to before and during the bloom (experiments 1, 3, and 5) (Additional file 6: Figure S6a–f). Interestingly, time zero samples indicated that representatives of the *Alteromonadales* order, which includes *Pseudoalteromonas*, associated with particles generally decreased in abundance over the course of the bloom, while free-living representatives increased during experiment 3 then decreased during experiment 5 and 7 (Fig. 6). Members of the *Alteromonadales* order are known to be associated with eukaryotic hosts [38, 39], and these associations may have been disrupted with the shift in the dominant eukaryotic phytoplankton and subsequent increase in cellular aggregates and fecal pellets over the course of the bloom. In the free-living fraction, a significant increase in the relative abundance of two *Pseudoalteromonas* ASVs corresponding to experiment 3 only was observed after HHQ treatment, indicating HHQ had a greater impact on *Pseudoalteromonas* spp. in particle-associated communities at multiple time points over the course of the bloom in comparison to free-living communities. Upon closer inspection of *Pseudoalteromonas* ASV_148, which significantly increased in relative abundance in both particle-associated and free-living fractions, we observed that in every instance when HHQ exposure experiments were performed, ASV_148 increased in relative abundance compared to the paired DMSO control, with significant increases occurring during the bloom phase (Fig. 8). Moreover, a similar trend was observed for all other *Pseudoalteromonas* ASVs (22, 49, 117) found to have significantly increased in response to HHQ at some point during the bloom, with relative abundances of these additional ASVs frequently higher under conditions when HHQ was present in comparison to DMSO and time zero controls (Additional file 6: Figure S6). These results indicate that in natural systems, marine *Pseudoalteromonas* spp. increase their relative abundance in response to nanomolar concentrations of an alkylquinolone signaling molecule they are known to produce. Of interest would be to investigate if *Pseudoalteromonas*’ predatory nature against competitive bacteria could be attributed to alkylquinolone signaling via binding partners (Additional file 10: Table S2) in response to exogenous HHQ application similar to what is seen in *P. aeruginosa.*

Indeed, alkylquinolones may help protect the *Pseudoalteromonas* particle-associated niche from competitors or enable opportunistic behavior to increase their growth relative to other members of the phycosphere community. In the biomedical literature, physiological concentrations (i.e., low micromolar) of HHQ have been shown to inhibit both bacterial motility and biofilm attachment/formation, as well as being bacteriostatic—exhibiting antimicrobial activity against a range of both Gram-negative and Gram-positive microorganisms [17]. Additionally, membrane vesicles containing large concentrations of alkylquinolones derived from *P. aeruginosa* strongly inhibit the growth of both Gram-positive and Gram-negative bacteria whereby these vesicles can fuse to bacterial competitors and release their contents [40]. Interestingly, the production of these membrane vesicles can be stimulated by exogenous application of alkylquinolones. Similar to *P. aeruginosa*, three strains of *Pseudoalteromonas piscicida* also produce extracellular vesicles of similar size capable of being transferred to the surface of *Vibrio* cells and inducing lysis [41]; however, the alkylquinolone content of these vesicles has not yet been investigated.

## Conclusions

Our findings have important implications for the ability of alkylquinolones such as HHQ to modify the composition of natural marine microbial communities. We demonstrate that nanomolar concentrations of the QS signal, HHQ were capable of decreasing the growth rate of photosynthetic eukaryotes based on bulk chlorophyll measurements; however, HHQ exposure did not significantly change the composition of the eukaryotic community. In contrast, heterotrophic prokaryote populations were restructured in the presence of HHQ, with the most notable finding that *Pseudoalteromonas* spp., a genus known to produce HHQ, significantly increased in relative abundance in both particle-associated and free-living bacterial communities. In addition, ASVs belonging to the genera *Psychrobacter*, *Sulfitobacter*, and *Glaciecola* were found to respond positively to HHQ incubations, and a mining of representative genomes from these groups indicates they contain putative binding partners for HHQ, suggesting these bacteria are capable of listening in and interpreting information from exogenous chemical signals in complex microbial communities. This study is the first to investigate how small molecule signals like alkylquinolones govern microbe-microbe interactions in heterogeneous environments in natural marine ecosystems and how these molecular interactions impact their eukaryotic hosts. Given the ubiquity of *Pseudoalteromonas* and *Pseudomonas* spp. in the marine environment, and the pervasiveness of naturally occurring alkylquinolones, our results suggest that there is potential for alkylquinolone signaling to play a role in structuring complex microbial communities. This study provides the framework upon which to start interrogating how chemical messages can influence both community homeostasis and intracellular interactions.

## Methods

### Mesocosm setup, sampling, and HHQ addition experiments

Samples were collected from mesocosm experiments conducted from 13 May through 30 May 2017 at the National Mesocosm Facility located at the Espeland Marine Biological Station at the Raune-fjord (60^o^22.1′N, 5^o^28.1′E), University of Bergen, Norway. Twelve polyethylene enclosures measuring 2 m diameter, 8 m deep, hereafter referred to as mesocosms, were moored to a raft approx. 200 m from shore. During assembly, approximately 20,000 L of unfiltered fjord seawater was enclosed in each mesocosm and monitored for 17 days. Three of the twelve mesocosms were amended on two consecutive days with pulses of inorganic nitrogen and phosphorus in Redfield ratio proportions in order to induce a phytoplankton bloom (total additions 4 μM nitrate, 0.25 μM phosphate; hereafter referred to as replete mesocosms). Mesocosms were bubbled with ambient air for two days after nutrient additions to facilitate mixing. Samples for nutrient analysis (N and P) were filtered through a combusted GF/F filter and stored at − 20 ^o^C. Nutrients were quantified using a Lachet QuickChem8500 Nutrient Analyzer Flow Injection Analysis System at the Rutgers Nutrient Analysis Facility. On May 15, nutrient measurements were determined to be 11.0 μM nitrate/nitrite and 1.4 μM phosphate. Mesocosm temperatures were monitored daily and average fluctuations at 1 m depth ranged from 10 to 11 ^o^C. Water was obtained from the replete mesocosms every two days over a 15-day period and either processed immediately for determination of chlorophyll *a* concentration, cell enumeration, and nucleic acid acquisition, or spiked with 410 nM (100 ng mL^−1^) 2-heptyl-4-quinolone (HHQ) or solvent vehicle controls and incubated for 24 h on land-based mesocosms. The HHQ concentration used in these experiments are based off the IC50 concentration for *E. huxleyi* reported in Harvey et al. (2016). On every experiment day, ~ 80 L of water from 1 m depth was collected via a 5-L Niskin bottle, passed through a 200-μm mesh filter to remove larger zooplankton, pooled in large carboys, and transported immediately to a 10 ^°^C cold room for further processing. Equal volumes were collected from the triplicate mesocosms. The water was then dispersed among nine, 4.7-L polycarbonate bottles that had been acid-washed and rinsed in 18.2 mΩ water (Millipore Milli-Q). Triplicate bottles representing time zero controls were immediately processed for determination of chlorophyll *a* concentration, cell enumeration, and nucleic acid acquisition (details below). The remaining bottles were amended in triplicate with either 410 nM (100 ng mL^−1^) HHQ dissolved in dimethyl sulfoxide (DMSO) or an equal concentration (0.1% *v*:*v*) of DMSO to serve as a solvent vehicle control. These six bottles were mixed well before incubation for 24 h in a land-based mesocosm containing flow-through surface seawater matching in situ temperatures. Window screen shading was used to replicate light levels corresponding to a depth of 1 m in the fjord-based mesocosms (7500 lx). The total time between subsampling the mesocosms and incubating the bottles was under 1 h. After a 24-h incubation, the bottles were recovered and processed in a 10 ^°^C cold room for determination of chlorophyll *a* concentration, cell enumeration, and for some experiments, nucleic acid acquisition as described below.

### Chlorophyll *a* measurements

Samples to determine chlorophyll *a* concentration were taken daily (for 17 days) from triplicate replete and control mesocosms at a depth of 1 m, and from HHQ and DMSO-spiked bottles after 24 h of incubation. Water samples (approx. 150 mL) were filtered under low vacuum pressure through 25-mm Whatman GF/F filters (effective pore size 0.7 μm). Filters were immediately extracted in vials containing 6 mL of 95% ethanol for 12–15 h in the dark at room temperature. Chlorophyll *a* concentrations were determined using a Turner TD700 fluorometer as previously described [42, 43]. Ethanol blanks were included, and samples were corrected for pheophytin using a drop of 10% hydrochloric acid prior to re-reading each sample.

### Cell enumeration by flow cytometry

Both phytoplankton and bacterial cell concentrations were determined daily from triplicate replete and control mesocosms using samples collected at a depth of 1 m, and from HHQ and DMSO-exposed communities after 24 h of incubation. Cell concentrations were determined from 200-μL samples using a Guava Technologies easyCyte BG HT flow cytometer (EMD Millipore). Samples were diluted in sterilized seawater to ensure < 500 cells μL^−1^ and avoid coincidence counting. Phytoplankton populations were resolved live based on their forward scatter, red (695/50), and yellow (575/25) emission parameters with 488-nm excitation. For bacteria/archaea enumeration, 5 mL of sample was preserved using glutaraldehyde (final concentration 0.5%) and frozen at − 80 ^°^C until analysis could be conducted (less than 2 months post experiment). Bacteria/archaea populations were resolved after staining samples with the DNA intercalating SYBR green I dye in the dark for at least 55 min based on their forward scatter and green (512/18) emission parameters with 488-nm excitation. Instrument-specific easyCheck beads were used for quality control and all samples were run at 0.24 μL s^−1^ for 3 min each. The abundance of three major phytoplankton groups (pico-, nano-, and *Synechococcus* spp.) determined by flow cytometry was converted to carbon biomass (μg C L^−1^) using published conservative conversion factors: 255 fg C cell^−1^ for *Synechococcus* [44], 1500 fg C cell^−1^ for picoeukaryotes [45], and 2763 fg C cell^−1^ for nanoeukaryotes, assuming an average cell diameter of 6 μm [46, 47].

Using both chlorophyll and cell abundance data, population changes in growth rate due to HHQ exposure were calculated using the exponential growth equation: growth rate = ln (*A*_24_/*A*_0_)/*T*_24_−*T*_0_ where *A* is abundance and *T* is time. Significant differences in growth rates of populations exposed to HHQ relative to the DMSO control were determined using a one-way analysis of variance (ANOVA) for each experiment. Alpha was set at *p* < 0.05 for all comparisons.

### Plankton identification

The relative abundance of various larger plankton assemblages (20–200 μm) at 1 m depth was analyzed at the start of every experiment using an automated FlowCam (Fluid Imaging Technologies). Samples were prepared and analyzed as previously described in [48]. In brief, 100–200 mL of sample was fixed in buffered formalin (final concentration 1% *v*:*v*) and stored at 4 ^°^C prior to analysis. Fixed samples were passed through a 200-μm mesh and ~ 90 mL was analyzed on Autoimage mode using a 300-μm flow cell and a flow rate of 2 mL min^−1^, for a particle capture efficiency of 39.7%. Approximately 5000–10,000 images were captured per sample and analyzed based on cell morphology.

### Biomass collection and DNA isolation

Triplicate microbial biomass samples from the HHQ addition experiments were taken at time zero (*T*_0_) directly from the pooled mesocosm sample and after 24 h (*T*_24_) exposure to either 410 nM HHQ (100 ng mL^−1^) or a DMSO (0.1 % *v*:*v*) solvent vehicle control. In a 10 ^°^C cold room, microbial biomass was harvested by passing between 0.8 and 2 L of sample through a 1-μm polycarbonate filter followed by a 0.2-μm polycarbonate filter via serial filtration with a peristatic pump. The microbial communities collected on these filters are referred to throughout as particle-associated (1–200 μm fraction) and free-living (0.2–1 μm fraction) communities. A peristaltic pump system fitted with silicon tubing and filter holders were flushed with 18.2 mΩ water (Millipore Milli-Q) between samples to prevent sample carry-over. Each sample was filtered in less than 30 min, then immediately placed in cryovials, flash frozen in liquid nitrogen, and stored at − 80 ^°^C until DNA isolation. DNA was isolated from 1 μm and 0.2 μm polycarbonate filters using an established protocol [49] with recent modifications [50], including steps to remove RNA contamination. Briefly, polycarbonate filters were thawed on ice and placed in lysing matrix E tubes containing 400 μL of phenol to chloroform to isoamyl alcohol (25:24:1, pH 8.0) and 400 μL of 2X TENS Buffer (100 mM Tris-HCl, pH 8.0, 40 mM EDTA, 200 mM NaCl, 2% SDS), agitated for 10 min using a horizontal vortex adapter, and centrifuged at 14,000 rpm for 6 min. The aqueous phase was carefully transferred to Phase Lock Gel (PLG) tubes (Quanta Bio) containing 375 μL chloroform, mixed via gentle inversion, and centrifuged at 14,000 rpm for 6 min. The supernatant was transferred to a sterile microcentrifuge tube and incubated with 0.5 μL of RNase A (100 mg/mL; Qiagen) at 37 ^°^C for 30 min after mixing by gentle inversion. After RNase treatment, samples were transferred to a new PLG tube containing 300 μL of 7.5 M ammonium acetate and mixed by gentle inversion before the addition of 700 μL of chloroform and additional mixing by inversion. These tubes were centrifuged at 14,000 rpm for 6 min, and the supernatant was transferred to a sterile microcentrifuge tube in which DNA was recovered by alcohol precipitation using 360 μL of ice-cold isopropanol containing 2 μL of linear acrylamide (5 mg mL^−1^; AMRESCO). Samples were mixed thoroughly by repeated inversions before incubating on ice for 1 h. DNA pellets were formed by centrifugation (14,800 rpm for 15 min at 4 ^o^C), at which point the isopropanol was removed and the DNA pellet was washed with 500 μL of ice-cold 75% ethanol. DNA pellets were again formed (14,800 rpm for 8 min at 4 ^o^C) before removing the ethanol by decanting and drying the pellets in a laminar flow hood for 2–5 min. Pellets were resuspended in 40 μL of nuclease-free water, and the total DNA yield was quantified using a NanoDrop 2000 spectrophotometer (Thermo Scientific) with yields ranging from 0.2–4.5 μg total DNA.

### Amplicon sequencing and analysis

Sequencing libraries were prepared and sequenced by the Georgia Genomics and Bioinformatics Core at the University of Georgia targeting the V4–V5 region of the 16S rRNA gene and the V9 region of the 18S rRNA gene. Libraries were sequenced using the Illumina MiSeq platform to produce 300 + 300 nt paired reads. Reads were quality trimmed and amplicon sequence variants (ASVs) identified using the DADA2 package (V1.9.1; (58)). Taxonomic assignments were made using the IDTAXA algorithm [51] and the training set for the SILVA small subunit rRNA database (release 132) within the DECIPHER package (V2.9.2; [52, 53]) for 16S rRNA gene reads, and a native implementation of the naïve Bayesian classifier method [54] within DADA2 using the training set for the Protist Ribosomal Reference database (PR2; V4.10.0) [55] for 18S reads. 16S rRNA gene reads identified as chloroplast sequences at the order level were further classified by BLASTn searches of the PhytoRef database downloaded September 2018 [56]. Diversity and community composition analyses were performed using the phyloseq package (V1.26.0; (69)) and differential abundances were determined using a combination of DESeq2 (V1.22.1; [57]) and EdgeR (V3.24.1; [58]) after comparisons using the DAtest package (V2.7.11; [59]). In order to comprehensively examine how microbial community composition was impacted by HHQ over the course of the bloom, *T*_0_ DNA samples from all experiments, and *T*_24_ DNA samples from experiments 1, 3, 5, and 7 were chosen to prepare 16S and 18S rRNA gene amplicon libraries for sequencing. Libraries targeting the V4–V5 region of the 16S rRNA gene were constructed using the following primers: 515F (5′-GTGYCAGCMGCCGCGGTAA-3′) and 926R (5′-CCGYCAATTYMTTTRAGTTT-3′) to obtain longer amplicons, reduce biases against archaea and the SAR11 clade, and obtain eukaryotic plastid sequences [60, 61]. Libraries targeting the V9 region of the 18S rRNA gene were constructed using the following primers: Euk1391F (5′-GTACACACCGCCCGTC-3′) and EukBr (5′-TGATCCTTCTGCAGGTTCACCTAC-3′) to target microbial eukaryotic lineages [62, 63]. PCR amplification was performed following the protocols and standards recommended by the Earth Microbiome Project for preparation of 16S and 18S rRNA amplicons for Illumina sequencing ([63]); earthmicrobiome.org), and libraries were prepared using procedures outlined in the Illumina 16S metagenomic sequencing library preparation guide [64] using an input of 25 ng of DNA. Amplicon libraries were multiplexed in two sets of 72 and sequenced using the Illumina MiSeq platform to produce 300 + 300 nt paired reads. After demultiplexing, three samples were found to contain anomalously low information (< 300 reads each) and were removed from further analysis. Of the remaining samples, a median total of approx. 150 K raw paired-end reads were obtained for each sample (range approx. 14 K—1.2 million due to variations in library loading).

### Read processing and identification of amplicon sequence variants

Processing of amplicon reads and figure generation were done using R (V3.5.1; https://www.R-project.org). Pre-processing of reads and inference of amplicon sequence variants (ASVs) was performed using the DADA2 package (V1.9.1), providing de novo identification of high-resolution exact sample sequences [65, 66]. After removal of primer sequence and inspection of read quality profiles, reads were truncated as follows to remove low-quality nucleotides while maintaining sufficient (> 20 nt) overlap between paired reads: 16S rRNA reads truncated at 245 nt (forward) and 195 nt (reverse); 18S rRNA reads truncated at 200 nt (forward) and 180 nt (reverse). Reads were filtered and phiX contamination removed using DADA2 standard filtering parameters. Error rates were determined using the first billion bases of each dataset prior to dereplication and inference of ASVs. Paired reads were then merged (ca. 99% of reads successfully merged) and reads with unexpected lengths (< 1% of reads) and chimeras (ca. 2% of reads) were identified and removed using the default DADA2 parameters.

The castor package (V1.3.4; [67]) was used to calculate the nearest sequenced taxon distance (NSTD) for each 16S rRNA ASV in our dataset compared to a set of 6780 16S rRNA gene sequences derived from closed bacterial and archaeal genomes downloaded from the NCBI RefSeq database described by [68] and compared to 16S rRNA gene sequences from reference genomes used by PICRUSt [69]. Reference sequences were aligned with our ASVs using MAFFT (V7.388), and phylogenetic trees were generated using RAxML (V8.2.11) with default settings in the Geneious software package (V10.2.2). NSTDs were averaged to determine the nearest sequenced taxon index (NSTI) for our study. The NSTI was found to be 23.9% and 23.8% when compared to the NCBI RefSeq and PICRUSt databases, respectively. Given these NSTI values and the fact that we do not focus our analyses on proportional changes of ASVs within a sample [70], we chose not to correct for 16S gene copy number to avoid introducing additional noise and to increase the comparability of our results to other amplicon sequencing studies based on the recommendations of [71].

### Core diversity and community composition analyses

Tables produced by the DADA2 pipeline were imported into the phyloseq package (V1.26.0; [71] to evaluate core diversity metrics and community composition. Rarefaction curves were produced using the ggrare function with default settings in the ranacapa package (Additional file 7: Figure S7). Prior to diversity analysis, 16S ASVs that could not be identified at the domain level and 18S ASVs that could not be identified at the supergroup level were removed. Chloroplast sequences in the 16S data further identified through a BLASTn search of the PhytoRef database were separated into a distinct phyloseq object. The remaining 16S taxa were further processed to separate ASVs identified as cyanobacteria at the phylum level. 18S taxa were filtered to remove heterotrophic protists and material derived from fragments of larger zooplankton (e.g., Metazoa). Each subset was pruned to remove ASVs that were no longer present. Taxa remaining in each subset were grouped at the order (16S) or division (18S) level, and the relative abundance of the top four (chloroplast reads) or five (all other subsets) groups (approx. 75–99% of the total identifiable community) was compared using the plot_bar function in phyloseq.

Tukey box plots displaying the Shannon alpha-diversity index were produced using the plot_richness function (Additional file 2: Figure S2c, d and Additional file 8: Figure S8). Singletons were removed from each subset and read counts were transformed to be proportional prior to ordination by non-metric multidimensional scaling using the Bray-Curtis distance metric. Ordinations were also constructed by principal coordinate analysis based on weighted UniFrac distances after aligning ASVs using the DECIPHER package (V2.9.2) and construction of phylogenetic trees using the phangorn package with default settings (V2.4.0; [72]). Significance testing of ordinations by PERMANOVA analysis was conducted with 999 permutations using the vegan package (V2.5.2; [73]).

### Differential relative abundance determination

Given the large number of methods currently available to determine differential relative abundance in amplicon sequence data and debates regarding their utility [74–77], we employed the DAtest package (V2.7.11; [59]) to identify which methods are most appropriate for our specific dataset. Samples were pre-processed to group low abundance ASVs (< 3 reads in < 3 samples) into a single aggregate feature using the preDA function. The testDA function was then run over a range of effect sizes to compare each method’s ability to identify differentially abundant ASVs (seed set to 123). Of the 25 methods tested, DESeq2 (V1.22.1; [57]) and EdgeR (V3.24.1; [58]) with relative log expression normalization consistently produced the highest values for the area under the receiver operating characteristic curves and spike detection rates and the lowest false positive and false discovery rates, particularly at higher effect sizes. Relative abundance differences between ASVs exposed to HHQ or a DMSO solvent control for 24 h were then calculated for all methods using the allDA function and ASVs identified as significant (BH-adjusted *p* value < 0.1, corresponding to ≤ 0.94 expected false positives) were compared among methods using the vennDA function. DESeq2 and EdgeR with relative log expression normalization were consistently the most conservative methods (i.e., identified the lowest number of significantly different ASVs), and we chose to focus our analyses on ASVs that exhibited significant changes in relative abundance according to both methods.

## Additional files

Additional file 1: **Figure S1.** Group-specific phytoplankton cell abundances and biomass over the course of the bloom. (DOCX 568 kb)

Additional file 2: **Figure S2.** Relative abundances of eukaryotic phytoplankton divisions (≥ 1%) determined by 18S rRNA with and without the inclusion of Dinoflagellata sequences and 16S chloroplast amplicon sequence variants. (DOCX 409 kb)

Additional file 3: **Figure S3.** Examination of cyanobacterial relative abundance over the course of the bloom and in response to HHQ exposure. (DOCX 312 kb)

Additional file 4: **Figure S4.** Amplicon sequence variants in particle-associated communities that significantly (log2 fold change) increased or decreased in relative abundance following 24 h exposure to HHQ compared to the DMSO solvent control. (DOCX 365 kb)

Additional file 5: **Figure S5.** Amplicon sequence variants in free-living communities that significantly (log2 fold change) increased or decreased in relative abundance following 24 h exposure to HHQ compared to the DMSO solvent control. (DOCX 492 kb)

Additional file 6: **Figure S6.** Normalized counts of bacterial ASVs at time zero and after 24 h exposure to HHQ or DMSO control. (DOCX 372 kb)

Additional file 7: **Figure S7.** Rarefaction curves of 18S and 16S rRNA amplicon library samples described in this study. (DOCX 840 kb)

Additional file 8: **Figure S8.** Tukey boxplots depicting the Shannon diversity index for 18S, 16S chloroplast, and heterotrophic bacteria that are particle-associated or free-living. (DOCX 314 kb)

Additional file 9: **Table S1.** Relative abundance of heterotrophic prokaryotes in particle-associated (> 1 μm) and free-living communities for orders representing ≥ 1% of the community in at least one sample. (DOCX 62 kb)

Additional file 10: **Table S2.** Genome mining of HHQ binding partners from bacterial representatives closely related to those ASVs that were significantly induced in response to HHQ exposure. (DOCX 31 kb)

## Data Availability

Chlorophyll and flow cytometry data are available via BCO-DMO database located at (https://www.bco-dmo.org/project/645515). No custom code was generated to process or analyze these data. Software versions and relevant parameters utilized are outlined within relevant sections of the methods. Sequences from this study are available at the NCBI SRA under BioProject ID PRJNA513038 (http://www.ncbi.nlm.nih.gov/bioproject/513038).
