# Supplementary material for: Bacterial alkylquinolone signaling contributes to structuring microbial communities in the ocean
Source: Microbiome. 2019 Jun 17;7:93. doi: 10.1186/s40168-019-0711-9 (PMC6580654; doi:10.1186/s40168-019-0711-9)
Supplement: Supplementary file 1 — Figure S1. Group-specific phytoplankton cell abundances and biomass over the course of the bloom. (DOCX 568 kb) [file 40168_2019_711_MOESM1_ESM.docx]

**
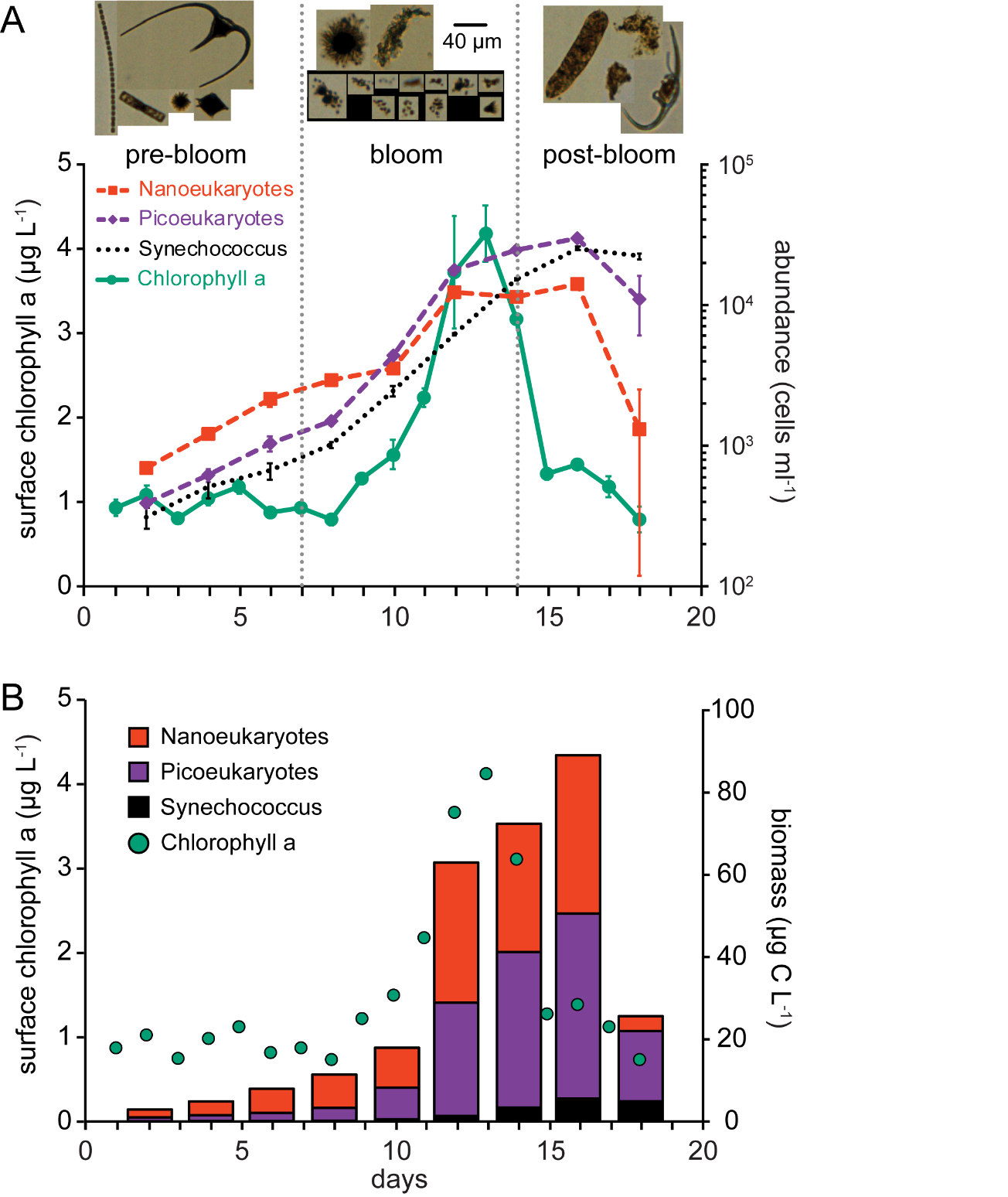
**

**Figure S1**. Group specific phytoplankton cell abundances and biomass over the course of the bloom. Cell concentrations nanoeukaryotic phytoplankton (squares; orange), picoeukaryotic phytoplankton (diamonds; purple) and *Synechococcus* (black dotted line) determined by flow cytometry shown alongside measurements of chlorophyll a (A). Symbols represent the mean (± s.d.) of biological triplicates. Representative images obtained using an automated FlowCam from various stages of the bloom are shown at the top of panel A. Biomass contributions of nanoeukaryotic phytoplankton (orange), picoeukaryotic phytoplankton (purple), and *Synechococcus* (black) calculated from the flow cytometry data using conversion factors as described in the methods. Chlorophyll a data are reproduced from Figure 1.
