# Supplementary material for: Bacterial alkylquinolone signaling contributes to structuring microbial communities in the ocean
Source: Microbiome. 2019 Jun 17;7:93. doi: 10.1186/s40168-019-0711-9 (PMC6580654; doi:10.1186/s40168-019-0711-9)

**Figure S2.** Relative abundances of eukaryotic phytoplankton divisions (≥ 1%) determined by 18S rRNA with (A) and without (B) inclusion of Dinoflagellata sequences and 16S chloroplast (C) amplicon sequence variants. X-axes indicate experimental time points and A and C are reproduced from Figure 2. Spearman’s rank correlation tests (bottom row) comparing the relative abundances determined by 18S rRNA and 16S chloroplast genes for all major eukaryotic phytoplankton divisions after removal of Dinoflagellata sequences from the 18S rRNA data set.


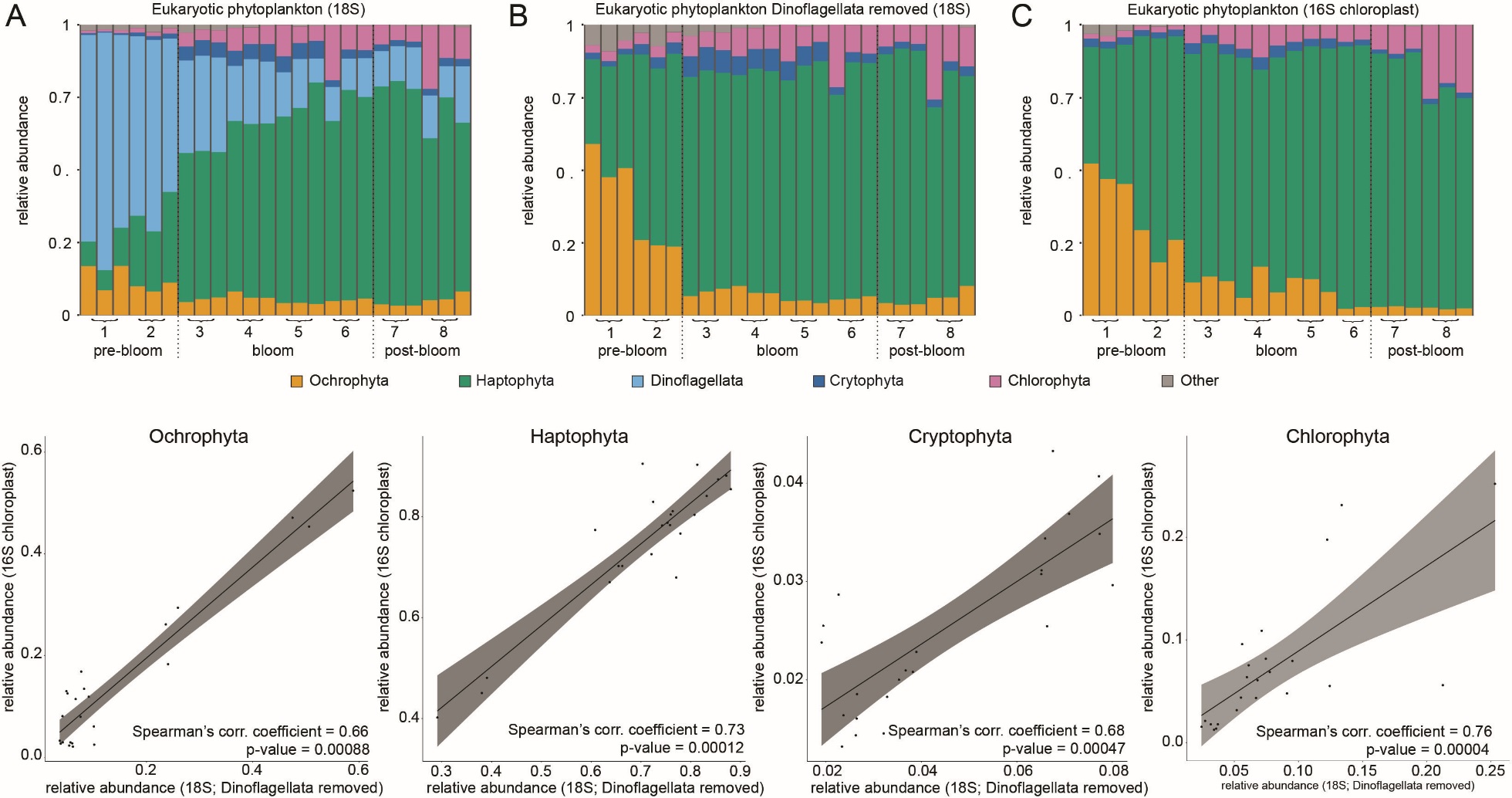

Supplement: Supplementary file 2 — Figure S2. Relative abundances of eukaryotic phytoplankton divisions (≥ 1%) determined by 18S rRNA with and without the inclusion of Dinoflagellata sequences and 16S chloroplast amplicon sequence variants. (DOCX 409 kb) [file 40168_2019_711_MOESM2_ESM.docx]
