# Supplementary material for: Bacterial alkylquinolone signaling contributes to structuring microbial communities in the ocean
Source: Microbiome. 2019 Jun 17;7:93. doi: 10.1186/s40168-019-0711-9 (PMC6580654; doi:10.1186/s40168-019-0711-9)
Supplement: Supplementary file 3 — Figure S3. Examination of cyanobacterial relative abundance over the course of the bloom and in response to HHQ exposure. (DOCX 312 kb) [file 40168_2019_711_MOESM3_ESM.docx]

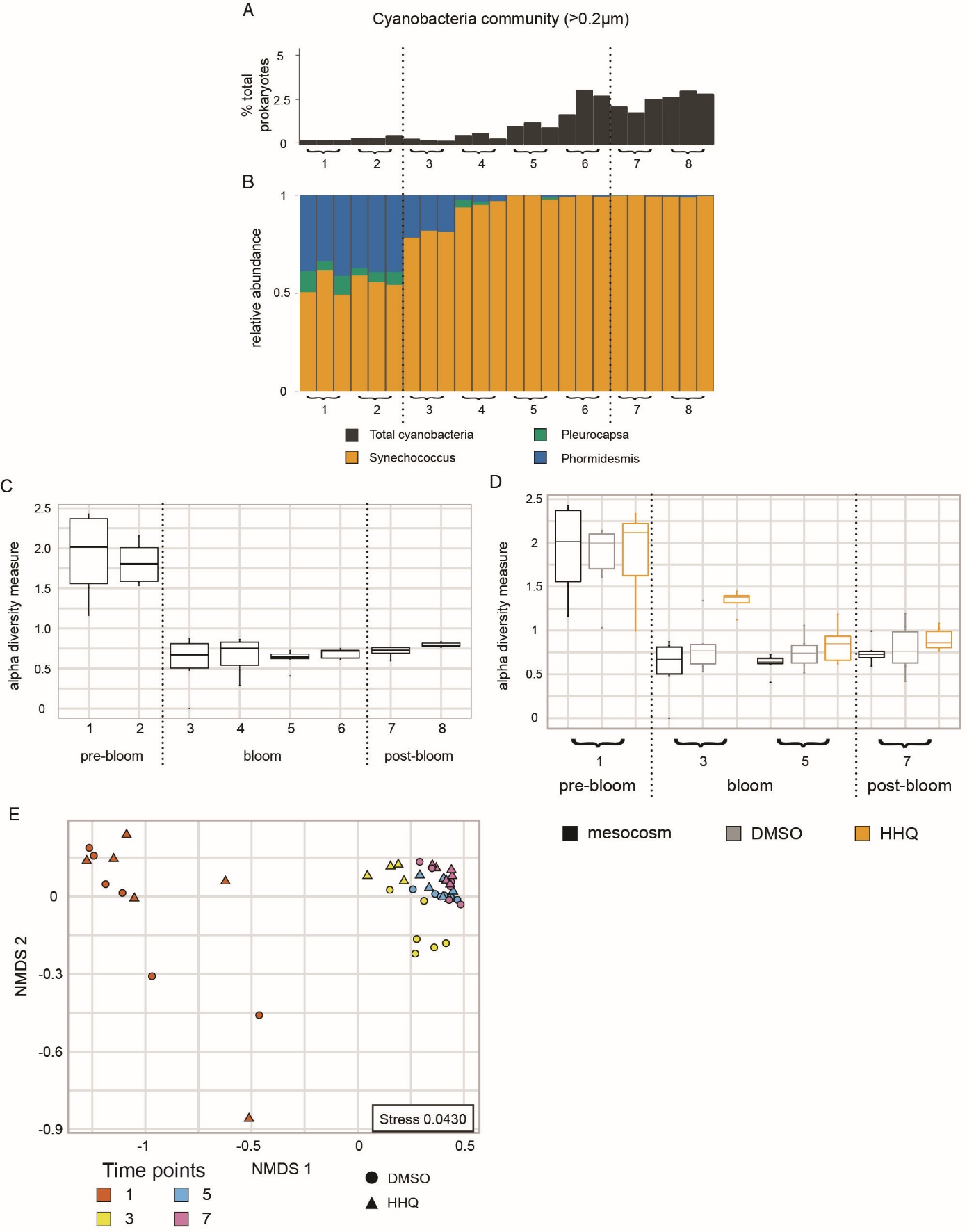


**Figure S3.** Percentage of the total prokaryotic community represented by cyanobacteria (A) and the relative contribution of different cyanobacteria genera to the total cyanobacteria community (B) determined by 16S amplicon sequence variants. Tukey boxplots depicting the Shannon diversity index for cyanobacteria communities sampled from the replete mesocosms over the eight time points noted in Figure 1 (C), and samples exposed for 24 hr (D) to either DMSO (grey) or 2-heyptyl-4-quinolone (HHQ; orange). Each boxplot is produced from a set of three to six discrete samples. Ordination of cyanobacteria communities based on 16S sequence variants visualized by non-metric multidimensional scaling (NMDS) of Bray-Curtis distance (E) with samples colored according to the sampling time points depicted in Figure 1 and representing communities exposed for 24 hr to either a DMSO solvent control (circles) or HHQ (triangles).
