# Supplementary material for: Bacterial alkylquinolone signaling contributes to structuring microbial communities in the ocean
Source: Microbiome. 2019 Jun 17;7:93. doi: 10.1186/s40168-019-0711-9 (PMC6580654; doi:10.1186/s40168-019-0711-9)
Supplement: Supplementary file 5 — Figure S5. Amplicon sequence variants in free-living communities that significantly (log2 fold change) increased or decreased in relative abundance following 24 h exposure to HHQ compared to the DMSO solvent control. (DOCX 492 kb) [file 40168_2019_711_MOESM5_ESM.docx]

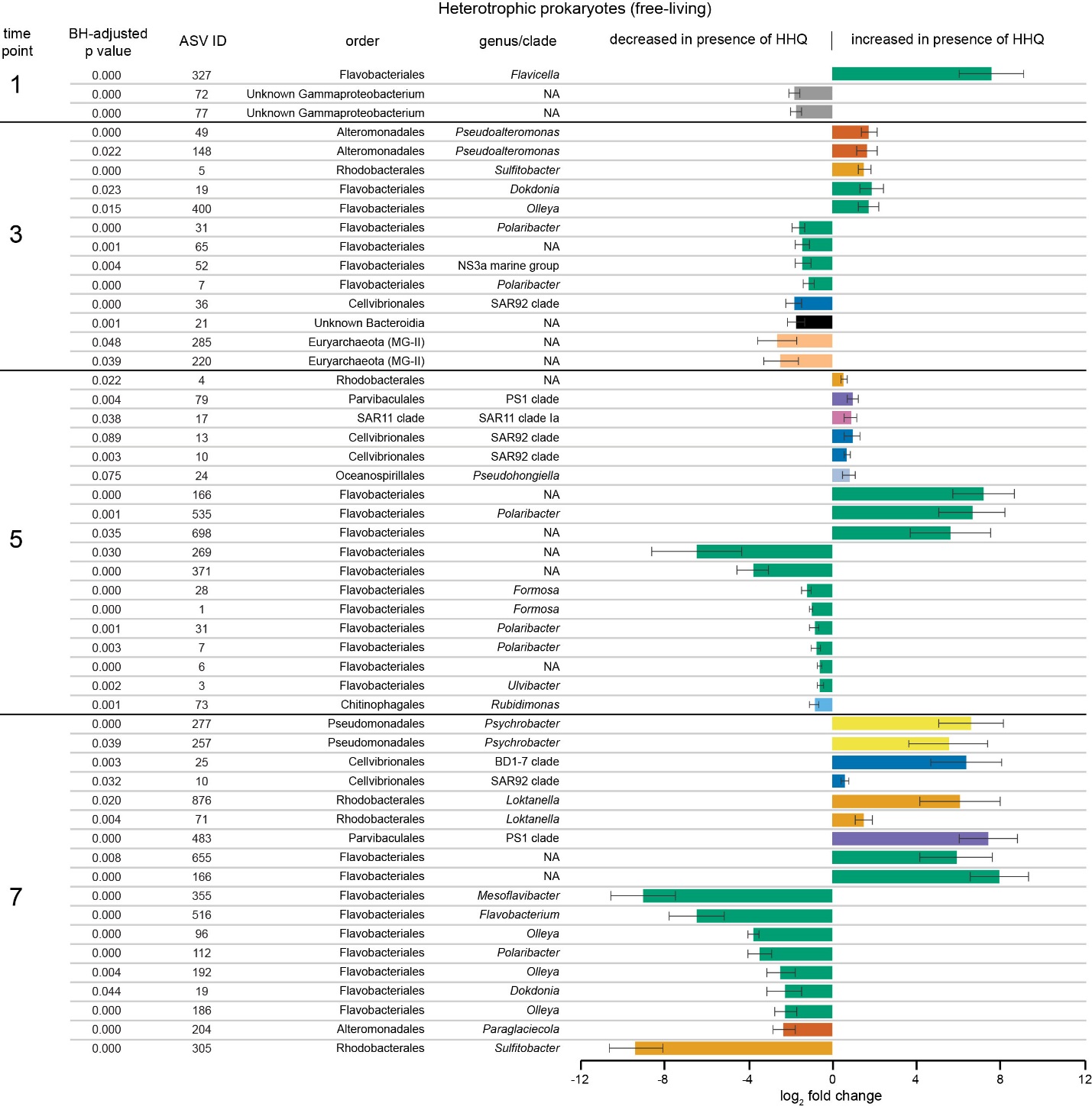


**Figure S5**. Amplicon sequence variants in free-living communities that significantly (log2 fold change) increased or decreased in relative abundance following 24 hr exposure to 410 nM (100 ng/mL) HHQ compared to the DMSO solvent control. Bars are colored by order and grouped according to the experimental time point and taxonomy. Time point numbers correspond to the sampling time points depicted in Figure 1. Significance was determined by BH-adjusted *p* values less than 0.1.
