# Supplementary material for: Bacterial alkylquinolone signaling contributes to structuring microbial communities in the ocean
Source: Microbiome. 2019 Jun 17;7:93. doi: 10.1186/s40168-019-0711-9 (PMC6580654; doi:10.1186/s40168-019-0711-9)
Supplement: Supplementary file 6 — Figure S6. Normalized counts of bacterial ASVs at time zero and after 24 h exposure to HHQ or DMSO control. (DOCX 372 kb) [file 40168_2019_711_MOESM6_ESM.docx]

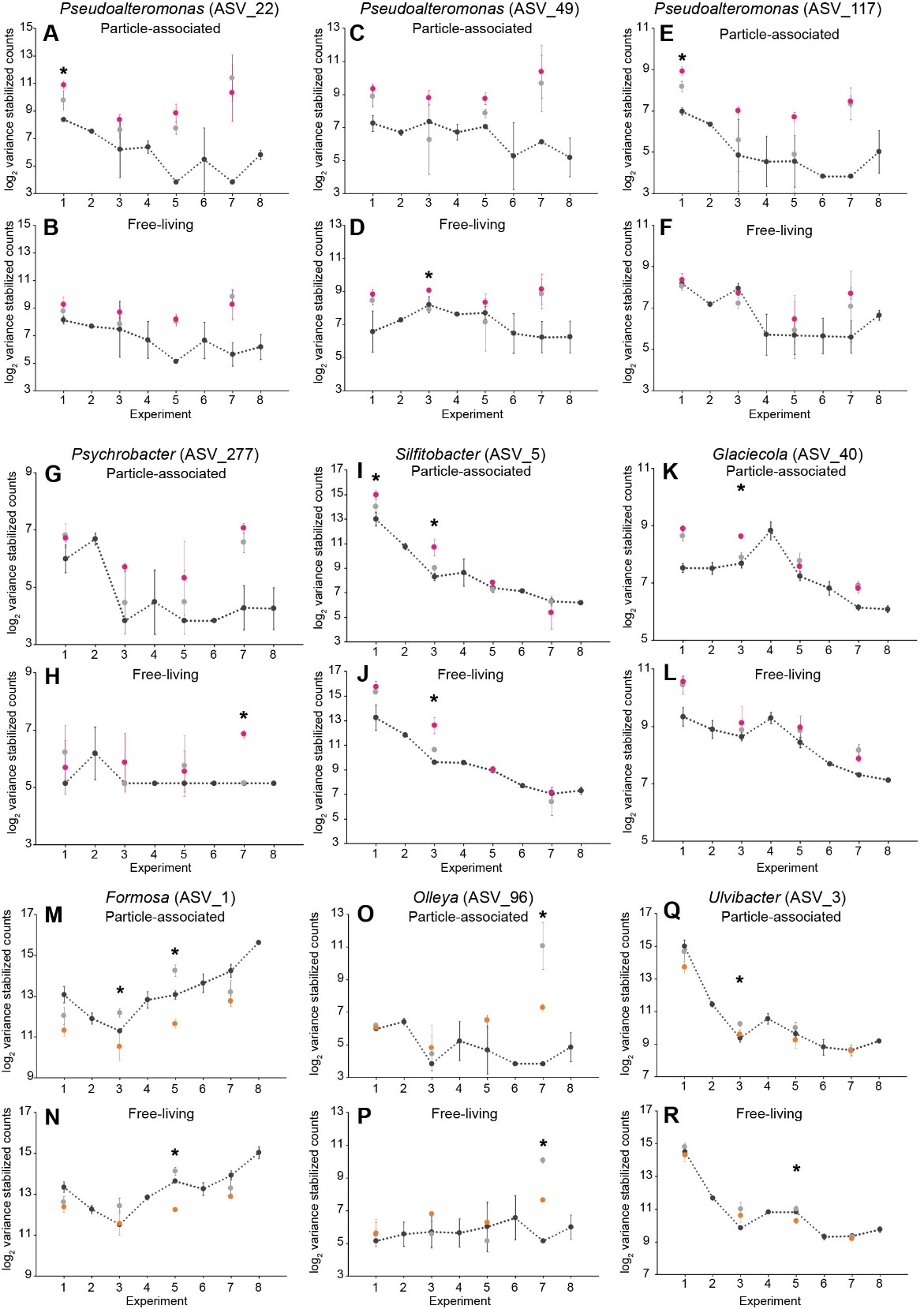


**Figure S6.** Normalized counts of bacterial ASVs in the replete mesocosms at time zero (black) and after 24 hr exposure to DMSO control (grey) or HHQ (magenta = increased; orange = decreased). Asterisks indicate a significant difference in relative abundance after HHQ exposure (BH-adjusted *p* value <0.1). Numbers along the X-axes correspond to the sampling time points described in Figure 1.
