# Supplementary material for: Bacterial alkylquinolone signaling contributes to structuring microbial communities in the ocean
Source: Microbiome. 2019 Jun 17;7:93. doi: 10.1186/s40168-019-0711-9 (PMC6580654; doi:10.1186/s40168-019-0711-9)
Supplement: Supplementary file 7 — Figure S7. Rarefaction curves of 18S and 16S rRNA amplicon library samples described in this study. (DOCX 840 kb) [file 40168_2019_711_MOESM7_ESM.docx]

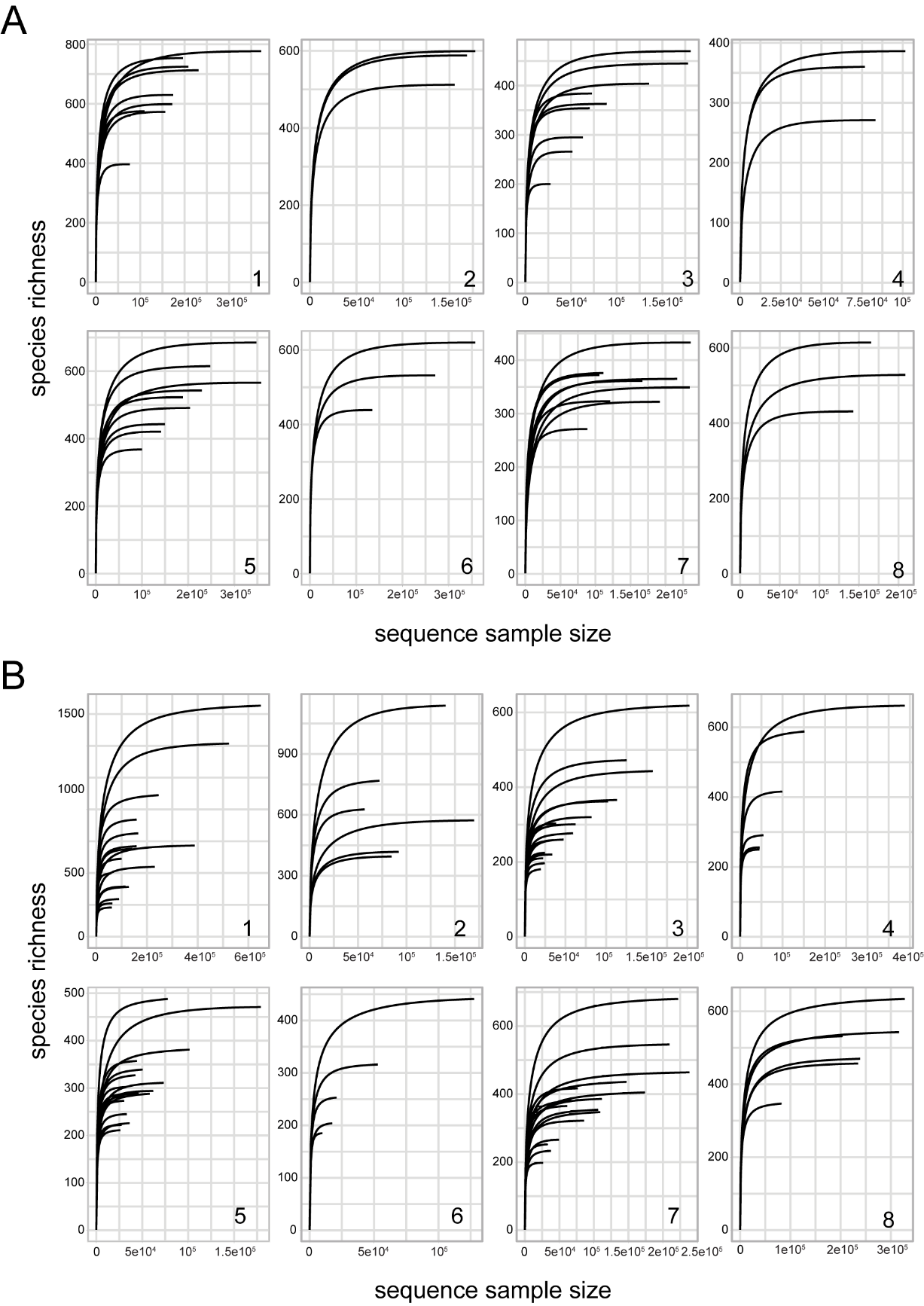


**Figure S7**. Rarefaction curves of 18S (A) and 16S (B) amplicon library samples described in this study. Curves are faceted according to the sampling time points described in Figure 1.
