# Supplementary material for: Bacterial alkylquinolone signaling contributes to structuring microbial communities in the ocean
Source: Microbiome. 2019 Jun 17;7:93. doi: 10.1186/s40168-019-0711-9 (PMC6580654; doi:10.1186/s40168-019-0711-9)
Supplement: Supplementary file 8 — Figure S8. Tukey boxplots depicting the Shannon diversity index for 18S, 16S chloroplast, and heterotrophic bacteria that are particle-associated or free-living. (DOCX 314 kb) [file 40168_2019_711_MOESM8_ESM.docx]

**
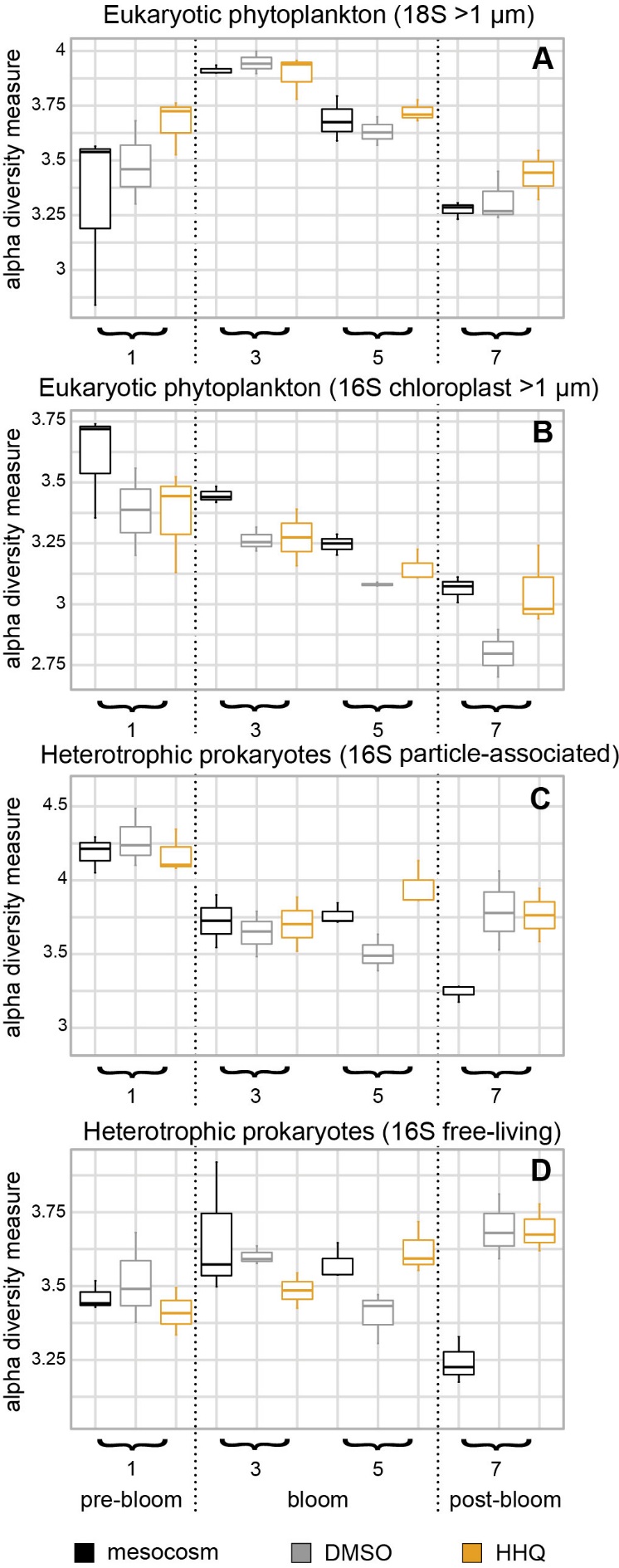
Figure S8.** Tukey boxplots depicting the Shannon diversity index for 18S (A), 16S chloroplast (B), and heterotrophic bacteria that are particle-associated (C) or free-living (D). Each boxplot is produced from a set of triplicate or duplicate samples. Boxplots are organized by bloom stage and colored to represent samples obtained directly from the replete mesocosms (black) or samples exposed to HHQ (orange) or DMSO control (grey) for 24 hours.
