# Supplementary material for: Bacterial alkylquinolone signaling contributes to structuring microbial communities in the ocean
Source: Microbiome. 2019 Jun 17;7:93. doi: 10.1186/s40168-019-0711-9 (PMC6580654; doi:10.1186/s40168-019-0711-9)
Supplement: Supplementary file 9 — Table S1. Relative abundance of heterotrophic prokaryotes in particle-associated (> 1 μm) and free-living communities for orders representing ≥ 1% of the community in at least one sample. (DOCX 62 kb) [file 40168_2019_711_MOESM9_ESM.docx]

**Table S1.** Relative abundance of heterotrophic prokaryotes in particle-associated (>1 µm) and free-living communities for orders representing ≥ 1% of the community in at least one sample.

| **particle-associated** |  |  |  |
| --- | --- | --- | --- |
| **order** | **treatment** | **experimental time point** | **relative abundance particle-associated** |
| Flavobacteriales | T0 | 1 | 0.485448518 |
| Rhodobacterales | T0 | 1 | 0.204859864 |
| Cellvibrionales | T0 | 1 | 0.085065847 |
| Alteromonadales | T0 | 1 | 0.04356647 |
| Unidentified | T0 | 1 | 0.045395936 |
| Pseudomonadales | T0 | 1 | 0.005810341 |
| Oceanospirillales | T0 | 1 | 0.001749613 |
| Chitinophagales | T0 | 1 | 0.015194472 |
| Betaproteobacteriales | T0 | 1 | 0.008914739 |
| Cytophagales | T0 | 1 | 0.017906309 |
| Sphingomonadales | T0 | 1 | 0.00405317 |
| Vibrionales | T0 | 1 | 0.01460389 |
| Rhizobiales | T0 | 1 | 0.020119807 |
| SAR11 clade | T0 | 1 | 0.000812185 |
| SAR116 clade | T0 | 1 | 6.00E-05 |
| Lactobacillales | T0 | 1 | 0.010166295 |
| Flavobacteriales | DMSO | 1 | 0.352251964 |
| Rhodobacterales | DMSO | 1 | 0.239395283 |
| Cellvibrionales | DMSO | 1 | 0.089927465 |
| Alteromonadales | DMSO | 1 | 0.091234778 |
| Unidentified | DMSO | 1 | 0.058147693 |
| Pseudomonadales | DMSO | 1 | 0.004782083 |
| Oceanospirillales | DMSO | 1 | 0.009296869 |
| Chitinophagales | DMSO | 1 | 0.014897093 |
| Betaproteobacteriales | DMSO | 1 | 0.008494586 |
| Cytophagales | DMSO | 1 | 0.033320903 |
| Sphingomonadales | DMSO | 1 | 0.006284032 |
| Vibrionales | DMSO | 1 | 0.0190879 |
| Rhizobiales | DMSO | 1 | 0.023293034 |
| SAR11 clade | DMSO | 1 | 0.000894536 |
| SAR116 clade | DMSO | 1 | 0.000123482 |
| Lactobacillales | DMSO | 1 | 0.01334249 |
| Flavobacteriales | HHQ | 1 | 0.313851231 |
| Rhodobacterales | HHQ | 1 | 0.300845039 |
| Cellvibrionales | HHQ | 1 | 0.08991867 |
| Alteromonadales | HHQ | 1 | 0.116583208 |
| Unidentified | HHQ | 1 | 0.032314889 |
| Pseudomonadales | HHQ | 1 | 0.00605634 |
| Oceanospirillales | HHQ | 1 | 0.012668239 |
| Chitinophagales | HHQ | 1 | 0.009395172 |
| Betaproteobacteriales | HHQ | 1 | 0.006054326 |
| Cytophagales | HHQ | 1 | 0.022102141 |
| Sphingomonadales | HHQ | 1 | 0.004588271 |
| Vibrionales | HHQ | 1 | 0.024688376 |
| Rhizobiales | HHQ | 1 | 0.017793196 |
| SAR11 clade | HHQ | 1 | 0.000544733 |
| SAR116 clade | HHQ | 1 | 2.05E-05 |
| Lactobacillales | HHQ | 1 | 0.014812318 |
| Flavobacteriales | T0 | 3 | 0.283395795 |
| Rhodobacterales | T0 | 3 | 0.201302688 |
| Cellvibrionales | T0 | 3 | 0.114008982 |
| Alteromonadales | T0 | 3 | 0.056819046 |
| Unidentified | T0 | 3 | 0.191296147 |
| Pseudomonadales | T0 | 3 | 0.045697258 |
| Oceanospirillales | T0 | 3 | 0.018038693 |
| Chitinophagales | T0 | 3 | 0.005932824 |
| Betaproteobacteriales | T0 | 3 | 0.01245316 |
| Cytophagales | T0 | 3 | 0.009171259 |
| Sphingomonadales | T0 | 3 | 0.00318639 |
| Vibrionales | T0 | 3 | 0.004901599 |
| Rhizobiales | T0 | 3 | 0.001676701 |
| SAR11 clade | T0 | 3 | 0.008459105 |
| SAR116 clade | T0 | 3 | 0.002891899 |
| Lactobacillales | T0 | 3 | 0.002686811 |
| Flavobacteriales | DMSO | 3 | 0.390532146 |
| Rhodobacterales | DMSO | 3 | 0.238506179 |
| Cellvibrionales | DMSO | 3 | 0.144290604 |
| Alteromonadales | DMSO | 3 | 0.039570792 |
| Unidentified | DMSO | 3 | 0.098007104 |
| Pseudomonadales | DMSO | 3 | 0.007509748 |
| Oceanospirillales | DMSO | 3 | 0.015023156 |
| Chitinophagales | DMSO | 3 | 0.014188483 |
| Betaproteobacteriales | DMSO | 3 | 0.014168978 |
| Cytophagales | DMSO | 3 | 0.005082822 |
| Sphingomonadales | DMSO | 3 | 0.00162565 |
| Vibrionales | DMSO | 3 | 0.001508539 |
| Rhizobiales | DMSO | 3 | 0.001537022 |
| SAR11 clade | DMSO | 3 | 0.003875916 |
| SAR116 clade | DMSO | 3 | 0.002107193 |
| Lactobacillales | DMSO | 3 | 0.000398449 |
| Flavobacteriales | HHQ | 3 | 0.228968865 |
| Rhodobacterales | HHQ | 3 | 0.404885545 |
| Cellvibrionales | HHQ | 3 | 0.125423387 |
| Alteromonadales | HHQ | 3 | 0.075182371 |
| Unidentified | HHQ | 3 | 0.06072269 |
| Pseudomonadales | HHQ | 3 | 0.012491814 |
| Oceanospirillales | HHQ | 3 | 0.016700689 |
| Chitinophagales | HHQ | 3 | 0.010453162 |
| Betaproteobacteriales | HHQ | 3 | 0.01964217 |
| Cytophagales | HHQ | 3 | 0.008422783 |
| Sphingomonadales | HHQ | 3 | 0.002011559 |
| Vibrionales | HHQ | 3 | 0.003313295 |
| Rhizobiales | HHQ | 3 | 0.002804397 |
| SAR11 clade | HHQ | 3 | 0.003418133 |
| SAR116 clade | HHQ | 3 | 0.001932976 |
| Lactobacillales | HHQ | 3 | 0.001569254 |
| Flavobacteriales | T0 | 5 | 0.462633093 |
| Rhodobacterales | T0 | 5 | 0.154642272 |
| Cellvibrionales | T0 | 5 | 0.108468097 |
| Alteromonadales | T0 | 5 | 0.028317078 |
| Unidentified | T0 | 5 | 0.08501597 |
| Pseudomonadales | T0 | 5 | 0.030684349 |
| Oceanospirillales | T0 | 5 | 0.01414266 |
| Chitinophagales | T0 | 5 | 0.02907409 |
| Betaproteobacteriales | T0 | 5 | 0.015247124 |
| Cytophagales | T0 | 5 | 0.007241467 |
| Sphingomonadales | T0 | 5 | 0.017709028 |
| Vibrionales | T0 | 5 | 0.003257649 |
| Rhizobiales | T0 | 5 | 0.00144542 |
| SAR11 clade | T0 | 5 | 0.007274484 |
| SAR116 clade | T0 | 5 | 0.003773503 |
| Lactobacillales | T0 | 5 | 0 |
| Flavobacteriales | DMSO | 5 | 0.557502311 |
| Rhodobacterales | DMSO | 5 | 0.137947269 |
| Cellvibrionales | DMSO | 5 | 0.081597716 |
| Alteromonadales | DMSO | 5 | 0.038358916 |
| Unidentified | DMSO | 5 | 0.082747365 |
| Pseudomonadales | DMSO | 5 | 0.013143105 |
| Oceanospirillales | DMSO | 5 | 0.015680047 |
| Chitinophagales | DMSO | 5 | 0.020878361 |
| Betaproteobacteriales | DMSO | 5 | 0.009030972 |
| Cytophagales | DMSO | 5 | 0.005798347 |
| Sphingomonadales | DMSO | 5 | 0.006585179 |
| Vibrionales | DMSO | 5 | 0.001109628 |
| Rhizobiales | DMSO | 5 | 0.000977258 |
| SAR11 clade | DMSO | 5 | 0.003375972 |
| SAR116 clade | DMSO | 5 | 0.003500498 |
| Lactobacillales | DMSO | 5 | 0.000209587 |
| Flavobacteriales | HHQ | 5 | 0.386937234 |
| Rhodobacterales | HHQ | 5 | 0.242920268 |
| Cellvibrionales | HHQ | 5 | 0.113126664 |
| Alteromonadales | HHQ | 5 | 0.073378888 |
| Unidentified | HHQ | 5 | 0.05023984 |
| Pseudomonadales | HHQ | 5 | 0.01658649 |
| Oceanospirillales | HHQ | 5 | 0.019871547 |
| Chitinophagales | HHQ | 5 | 0.02707621 |
| Betaproteobacteriales | HHQ | 5 | 0.015452565 |
| Cytophagales | HHQ | 5 | 0.008706686 |
| Sphingomonadales | HHQ | 5 | 0.006684858 |
| Vibrionales | HHQ | 5 | 0.002270826 |
| Rhizobiales | HHQ | 5 | 0.002020133 |
| SAR11 clade | HHQ | 5 | 0.003645634 |
| SAR116 clade | HHQ | 5 | 0.003491363 |
| Lactobacillales | HHQ | 5 | 0.000319835 |
| Flavobacteriales | T0 | 7 | 0.415873712 |
| Rhodobacterales | T0 | 7 | 0.261102278 |
| Cellvibrionales | T0 | 7 | 0.087715284 |
| Alteromonadales | T0 | 7 | 0.008200978 |
| Unidentified | T0 | 7 | 0.088874047 |
| Pseudomonadales | T0 | 7 | 0.010812976 |
| Oceanospirillales | T0 | 7 | 0.015374101 |
| Chitinophagales | T0 | 7 | 0.007444968 |
| Betaproteobacteriales | T0 | 7 | 0.011961801 |
| Cytophagales | T0 | 7 | 0.002701754 |
| Sphingomonadales | T0 | 7 | 0.012877889 |
| Vibrionales | T0 | 7 | 0.000354627 |
| Rhizobiales | T0 | 7 | 0.001734906 |
| SAR11 clade | T0 | 7 | 0.020843879 |
| SAR116 clade | T0 | 7 | 0.015011059 |
| Lactobacillales | T0 | 7 | 8.62E-06 |
| Flavobacteriales | DMSO | 7 | 0.543466252 |
| Rhodobacterales | DMSO | 7 | 0.151761218 |
| Cellvibrionales | DMSO | 7 | 0.054523587 |
| Alteromonadales | DMSO | 7 | 0.137637883 |
| Unidentified | DMSO | 7 | 0.025415682 |
| Pseudomonadales | DMSO | 7 | 0.015220793 |
| Oceanospirillales | DMSO | 7 | 0.011660812 |
| Chitinophagales | DMSO | 7 | 0.005047314 |
| Betaproteobacteriales | DMSO | 7 | 0.004509898 |
| Cytophagales | DMSO | 7 | 0.001161702 |
| Sphingomonadales | DMSO | 7 | 0.009623414 |
| Vibrionales | DMSO | 7 | 0.000845211 |
| Rhizobiales | DMSO | 7 | 0.002245248 |
| SAR11 clade | DMSO | 7 | 0.007639748 |
| SAR116 clade | DMSO | 7 | 0.00809861 |
| Lactobacillales | DMSO | 7 | 0 |
| Flavobacteriales | HHQ | 7 | 0.336187271 |
| Rhodobacterales | HHQ | 7 | 0.231514453 |
| Cellvibrionales | HHQ | 7 | 0.07846896 |
| Alteromonadales | HHQ | 7 | 0.21714306 |
| Unidentified | HHQ | 7 | 0.031307198 |
| Pseudomonadales | HHQ | 7 | 0.01600628 |
| Oceanospirillales | HHQ | 7 | 0.018914901 |
| Chitinophagales | HHQ | 7 | 0.004402323 |
| Betaproteobacteriales | HHQ | 7 | 0.007588542 |
| Cytophagales | HHQ | 7 | 0.001779745 |
| Sphingomonadales | HHQ | 7 | 0.004258852 |
| Vibrionales | HHQ | 7 | 0.001814229 |
| Rhizobiales | HHQ | 7 | 0.00104616 |
| SAR11 clade | HHQ | 7 | 0.012351761 |
| SAR116 clade | HHQ | 7 | 0.009894562 |
| Lactobacillales | HHQ | 7 | 0.000298021 |
|  |  |  |  |
|  |  |  |  |
| **free-living** |  |  |  |
| **order** | **treatment** | **experimental time point** | **relative abundance free-living** |
| Flavobacteriales | T0 | 1 | 0.507115557 |
| Rhodobacterales | T0 | 1 | 0.220188646 |
| Unidentified | T0 | 1 | 0.121800102 |
| Cellvibrionales | T0 | 1 | 0.085288062 |
| Alteromonadales | T0 | 1 | 0.017700816 |
| SAR11 clade | T0 | 1 | 0.006467708 |
| Betaproteobacteriales | T0 | 1 | 0.007093765 |
| Oceanospirillales | T0 | 1 | 0.003264206 |
| SAR116 clade | T0 | 1 | 0.000509137 |
| Micrococcales | T0 | 1 | 0.003908861 |
| Pseudomonadales | T0 | 1 | 0.00113652 |
| Tenderiales | T0 | 1 | 0 |
| Cytophagales | T0 | 1 | 0.007422352 |
| Parvibaculales | T0 | 1 | 0.001350809 |
| Sphingomonadales | T0 | 1 | 0.000650235 |
| SAR86 clade | T0 | 1 | 0.000504631 |
| Marine Group II Euryarchaeota | T0 | 1 | 0.002180352 |
| Vibrionales | T0 | 1 | 0.001844692 |
| Flavobacteriales | DMSO | 1 | 0.390469248 |
| Rhodobacterales | DMSO | 1 | 0.321527838 |
| Unidentified | DMSO | 1 | 0.099651496 |
| Cellvibrionales | DMSO | 1 | 0.089700037 |
| Alteromonadales | DMSO | 1 | 0.036932996 |
| SAR11 clade | DMSO | 1 | 0.004816394 |
| Betaproteobacteriales | DMSO | 1 | 0.008938585 |
| Oceanospirillales | DMSO | 1 | 0.01761971 |
| SAR116 clade | DMSO | 1 | 0.000620599 |
| Micrococcales | DMSO | 1 | 0.003772408 |
| Pseudomonadales | DMSO | 1 | 0.000423399 |
| Tenderiales | DMSO | 1 | 1.93E-05 |
| Cytophagales | DMSO | 1 | 0.004650375 |
| Parvibaculales | DMSO | 1 | 0.001803962 |
| Sphingomonadales | DMSO | 1 | 0.000484753 |
| SAR86 clade | DMSO | 1 | 0.000429212 |
| Marine Group II Euryarchaeota | DMSO | 1 | 0.001187982 |
| Vibrionales | DMSO | 1 | 0.006412402 |
| Flavobacteriales | HHQ | 1 | 0.381633136 |
| Rhodobacterales | HHQ | 1 | 0.348875239 |
| Unidentified | HHQ | 1 | 0.066093103 |
| Cellvibrionales | HHQ | 1 | 0.089856033 |
| Alteromonadales | HHQ | 1 | 0.04794282 |
| SAR11 clade | HHQ | 1 | 0.003881862 |
| Betaproteobacteriales | HHQ | 1 | 0.007407519 |
| Oceanospirillales | HHQ | 1 | 0.023214671 |
| SAR116 clade | HHQ | 1 | 0.000343622 |
| Micrococcales | HHQ | 1 | 0.004068942 |
| Pseudomonadales | HHQ | 1 | 0.000424733 |
| Tenderiales | HHQ | 1 | 7.73E-06 |
| Cytophagales | HHQ | 1 | 0.004303524 |
| Parvibaculales | HHQ | 1 | 0.001283815 |
| Sphingomonadales | HHQ | 1 | 0.000303442 |
| SAR86 clade | HHQ | 1 | 0.000505674 |
| Marine Group II Euryarchaeota | HHQ | 1 | 0.000449976 |
| Vibrionales | HHQ | 1 | 0.010897459 |
| Flavobacteriales | T0 | 3 | 0.246319852 |
| Rhodobacterales | T0 | 3 | 0.232508844 |
| Unidentified | T0 | 3 | 0.13034268 |
| Cellvibrionales | T0 | 3 | 0.10055764 |
| Alteromonadales | T0 | 3 | 0.13761887 |
| SAR11 clade | T0 | 3 | 0.0185049 |
| Betaproteobacteriales | T0 | 3 | 0.015056478 |
| Oceanospirillales | T0 | 3 | 0.011940132 |
| SAR116 clade | T0 | 3 | 0.004029348 |
| Micrococcales | T0 | 3 | 0.004784839 |
| Pseudomonadales | T0 | 3 | 0.034498934 |
| Tenderiales | T0 | 3 | 3.84E-05 |
| Cytophagales | T0 | 3 | 0.014836706 |
| Parvibaculales | T0 | 3 | 0.011109296 |
| Sphingomonadales | T0 | 3 | 0.005178874 |
| SAR86 clade | T0 | 3 | 0.001307635 |
| Marine Group II Euryarchaeota | T0 | 3 | 0.011553888 |
| Vibrionales | T0 | 3 | 0.001817538 |
| Flavobacteriales | DMSO | 3 | 0.359950883 |
| Rhodobacterales | DMSO | 3 | 0.306167123 |
| Unidentified | DMSO | 3 | 0.094650341 |
| Cellvibrionales | DMSO | 3 | 0.115133243 |
| Alteromonadales | DMSO | 3 | 0.02443602 |
| SAR11 clade | DMSO | 3 | 0.010669621 |
| Betaproteobacteriales | DMSO | 3 | 0.017220958 |
| Oceanospirillales | DMSO | 3 | 0.011491088 |
| SAR116 clade | DMSO | 3 | 0.003961011 |
| Micrococcales | DMSO | 3 | 0.006347591 |
| Pseudomonadales | DMSO | 3 | 0.004522879 |
| Tenderiales | DMSO | 3 | 0.000188886 |
| Cytophagales | DMSO | 3 | 0.009799487 |
| Parvibaculales | DMSO | 3 | 0.010392569 |
| Sphingomonadales | DMSO | 3 | 0.001520187 |
| SAR86 clade | DMSO | 3 | 0.001398268 |
| Marine Group II Euryarchaeota | DMSO | 3 | 0.004628713 |
| Vibrionales | DMSO | 3 | 0.000605235 |
| Flavobacteriales | HHQ | 3 | 0.256919632 |
| Rhodobacterales | HHQ | 3 | 0.464638559 |
| Unidentified | HHQ | 3 | 0.057286499 |
| Cellvibrionales | HHQ | 3 | 0.09194172 |
| Alteromonadales | HHQ | 3 | 0.038948166 |
| SAR11 clade | HHQ | 3 | 0.006231644 |
| Betaproteobacteriales | HHQ | 3 | 0.022781522 |
| Oceanospirillales | HHQ | 3 | 0.007742604 |
| SAR116 clade | HHQ | 3 | 0.002237583 |
| Micrococcales | HHQ | 3 | 0.009508234 |
| Pseudomonadales | HHQ | 3 | 0.006447027 |
| Tenderiales | HHQ | 3 | 0.000255477 |
| Cytophagales | HHQ | 3 | 0.00879242 |
| Parvibaculales | HHQ | 3 | 0.006099467 |
| Sphingomonadales | HHQ | 3 | 0.001233921 |
| SAR86 clade | HHQ | 3 | 0.000796367 |
| Marine Group II Euryarchaeota | HHQ | 3 | 0.000746335 |
| Vibrionales | HHQ | 3 | 0.001618524 |
| Flavobacteriales | T0 | 5 | 0.470189756 |
| Rhodobacterales | T0 | 5 | 0.212615277 |
| Unidentified | T0 | 5 | 0.102209672 |
| Cellvibrionales | T0 | 5 | 0.054220103 |
| Alteromonadales | T0 | 5 | 0.019286288 |
| SAR11 clade | T0 | 5 | 0.01408465 |
| Betaproteobacteriales | T0 | 5 | 0.027179789 |
| Oceanospirillales | T0 | 5 | 0.01214498 |
| SAR116 clade | T0 | 5 | 0.00568456 |
| Micrococcales | T0 | 5 | 0.010635044 |
| Pseudomonadales | T0 | 5 | 0.010595623 |
| Tenderiales | T0 | 5 | 0.005754993 |
| Cytophagales | T0 | 5 | 0.007990697 |
| Parvibaculales | T0 | 5 | 0.004104828 |
| Sphingomonadales | T0 | 5 | 0.015465355 |
| SAR86 clade | T0 | 5 | 0.002209303 |
| Marine Group II Euryarchaeota | T0 | 5 | 0.003936293 |
| Vibrionales | T0 | 5 | 0.000671759 |
| Flavobacteriales | DMSO | 5 | 0.512022594 |
| Rhodobacterales | DMSO | 5 | 0.231790758 |
| Unidentified | DMSO | 5 | 0.086516593 |
| Cellvibrionales | DMSO | 5 | 0.043292806 |
| Alteromonadales | DMSO | 5 | 0.020731433 |
| SAR11 clade | DMSO | 5 | 0.011253272 |
| Betaproteobacteriales | DMSO | 5 | 0.022102103 |
| Oceanospirillales | DMSO | 5 | 0.010357571 |
| SAR116 clade | DMSO | 5 | 0.005881834 |
| Micrococcales | DMSO | 5 | 0.006601696 |
| Pseudomonadales | DMSO | 5 | 0.005929271 |
| Tenderiales | DMSO | 5 | 0.00826107 |
| Cytophagales | DMSO | 5 | 0.004774408 |
| Parvibaculales | DMSO | 5 | 0.00499077 |
| Sphingomonadales | DMSO | 5 | 0.00328272 |
| SAR86 clade | DMSO | 5 | 0.00198779 |
| Marine Group II Euryarchaeota | DMSO | 5 | 0.002188854 |
| Vibrionales | DMSO | 5 | 0.000238573 |
| Flavobacteriales | HHQ | 5 | 0.344354514 |
| Rhodobacterales | HHQ | 5 | 0.320214968 |
| Unidentified | HHQ | 5 | 0.091291072 |
| Cellvibrionales | HHQ | 5 | 0.064326701 |
| Alteromonadales | HHQ | 5 | 0.032998046 |
| SAR11 clade | HHQ | 5 | 0.020894502 |
| Betaproteobacteriales | HHQ | 5 | 0.030089805 |
| Oceanospirillales | HHQ | 5 | 0.019311226 |
| SAR116 clade | HHQ | 5 | 0.007229107 |
| Micrococcales | HHQ | 5 | 0.007165269 |
| Pseudomonadales | HHQ | 5 | 0.006092787 |
| Tenderiales | HHQ | 5 | 0.01085827 |
| Cytophagales | HHQ | 5 | 0.008451337 |
| Parvibaculales | HHQ | 5 | 0.010206069 |
| Sphingomonadales | HHQ | 5 | 0.002494808 |
| SAR86 clade | HHQ | 5 | 0.004333802 |
| Marine Group II Euryarchaeota | HHQ | 5 | 0.001722703 |
| Vibrionales | HHQ | 5 | 0.00063573 |
| Flavobacteriales | T0 | 7 | 0.35459929 |
| Rhodobacterales | T0 | 7 | 0.304377732 |
| Unidentified | T0 | 7 | 0.086418847 |
| Cellvibrionales | T0 | 7 | 0.05749989 |
| Alteromonadales | T0 | 7 | 0.005200246 |
| SAR11 clade | T0 | 7 | 0.046144357 |
| Betaproteobacteriales | T0 | 7 | 0.022518452 |
| Oceanospirillales | T0 | 7 | 0.012922316 |
| SAR116 clade | T0 | 7 | 0.021837277 |
| Micrococcales | T0 | 7 | 0.010285086 |
| Pseudomonadales | T0 | 7 | 0.005099973 |
| Tenderiales | T0 | 7 | 0.021953939 |
| Cytophagales | T0 | 7 | 0.003730749 |
| Parvibaculales | T0 | 7 | 0.006511356 |
| Sphingomonadales | T0 | 7 | 0.012213948 |
| SAR86 clade | T0 | 7 | 0.008669309 |
| Marine Group II Euryarchaeota | T0 | 7 | 0.000430658 |
| Vibrionales | T0 | 7 | 7.45E-05 |
| Flavobacteriales | DMSO | 7 | 0.485818107 |
| Rhodobacterales | DMSO | 7 | 0.182540541 |
| Unidentified | DMSO | 7 | 0.042941785 |
| Cellvibrionales | DMSO | 7 | 0.064986413 |
| Alteromonadales | DMSO | 7 | 0.05890347 |
| SAR11 clade | DMSO | 7 | 0.041487234 |
| Betaproteobacteriales | DMSO | 7 | 0.014331543 |
| Oceanospirillales | DMSO | 7 | 0.014913188 |
| SAR116 clade | DMSO | 7 | 0.019456318 |
| Micrococcales | DMSO | 7 | 0.011005904 |
| Pseudomonadales | DMSO | 7 | 0.005828734 |
| Tenderiales | DMSO | 7 | 0.016196181 |
| Cytophagales | DMSO | 7 | 0.001153121 |
| Parvibaculales | DMSO | 7 | 0.006931727 |
| Sphingomonadales | DMSO | 7 | 0.006151456 |
| SAR86 clade | DMSO | 7 | 0.009375339 |
| Marine Group II Euryarchaeota | DMSO | 7 | 0 |
| Vibrionales | DMSO | 7 | 0.000470719 |
| Flavobacteriales | HHQ | 7 | 0.314093722 |
| Rhodobacterales | HHQ | 7 | 0.261520103 |
| Unidentified | HHQ | 7 | 0.05331866 |
| Cellvibrionales | HHQ | 7 | 0.094197492 |
| Alteromonadales | HHQ | 7 | 0.081650578 |
| SAR11 clade | HHQ | 7 | 0.048464864 |
| Betaproteobacteriales | HHQ | 7 | 0.020440609 |
| Oceanospirillales | HHQ | 7 | 0.020915669 |
| SAR116 clade | HHQ | 7 | 0.022222946 |
| Micrococcales | HHQ | 7 | 0.010474343 |
| Pseudomonadales | HHQ | 7 | 0.005023645 |
| Tenderiales | HHQ | 7 | 0.022427189 |
| Cytophagales | HHQ | 7 | 0.001424976 |
| Parvibaculales | HHQ | 7 | 0.010590624 |
| Sphingomonadales | HHQ | 7 | 0.003314479 |
| SAR86 clade | HHQ | 7 | 0.011198495 |
| Marine Group II Euryarchaeota | HHQ | 7 | 1.32E-05 |
| Vibrionales | HHQ | 7 | 0.000867559 |
