# Supplementary material for: Bacterial alkylquinolone signaling contributes to structuring microbial communities in the ocean
Source: Microbiome. 2019 Jun 17;7:93. doi: 10.1186/s40168-019-0711-9 (PMC6580654; doi:10.1186/s40168-019-0711-9)
Supplement: Supplementary file 10 — Table S2. Genome mining of HHQ binding partners from bacterial representatives closely related to those ASVs that were significantly induced in response to HHQ exposure. (DOCX 31 kb) [file 40168_2019_711_MOESM10_ESM.docx]

**Table S2**. Genome mining of HHQ binding partners from bacterial representatives closely related to those ASVs that were significantly induced in response to HHQ exposure.

|  |  |  | Bacterial species | | | | | | | | | | | | | |
| --- | --- | --- | --- | --- | --- | --- | --- | --- | --- | --- | --- | --- | --- | --- | --- | --- |
|  |  |  | *Sulfitobacter mediterraneus*KCTC 32188 | | *Sulfitobacter marinus* DSM 23422 | | *Sulfitobacter indolifex* HEL-45 | | *Sulfitobacter donghicola* KCTC 12864 | | *Glaciecola sp.* HTCC2999 | | *Psychrobacter fozii* | | *Pseudoalteromonas piscicida* A757 | |
|  |  | ASV ID; Percent identity --> | ASV_5, 99.4% identical | | ASV_5, 99.4% identical | | ASV_5, 99.4% identical | | ASV_5, 99.4% identical | | ASV_40, 99.4% identical | | ASV_257/277/461/855, 98.6% identical | | *n/a* | |
| **Gene function** | **Gene ID^a^** | **Genbank Accession number** | % ID | e-value | % ID | e-value | % ID | e-value | % ID | e-value | % ID | e-value | % ID | e-value | % ID | e-value |
| LysR-type regulator | *pqsR* | KT879198 | 26.7 | 2.21E-14 | 23.6 | 1.95E-08 | 27 | 1.53E-13 | 25.4 | 5.00E-17 | 23.3 | 7.69E-14 | 37.2 | 6.49E-52 | n/a | n/a |
| UDP-N-acetyl-2-amino-2-deoxy-D-glucuronate oxidase | *WbpB* | NP_251848.1 | 29.4 | 9.59E-12 | 21.1 | 5.07E-07 | no hit | no hit | no hit | no hit | 22.7 | 3.14E-04 | 55.4 | 1.14E-113 | 23.3 | 1.45E-02 |
| prokaryotic homolog to eukaryotic tubulin | *FtsZ* | NP_253097.1 | 53.3 | 3.07E-73 | 52.9 | 6.71E-74 | 52.2 | 2.37E-68 | 52.6 | 3.68E-78 | 61.2 | 2.45E-126 | 53.8 | 2.16E-84 | 56 | 6.34E-111 |
| N-succinylarginine dihydrolase | *AstB* | NP_249590.1 | no hit | no hit | no hit | no hit | no hit | no hit | no hit | no hit | 63.7 | 0.00E+00 | no hit | no hit | 60.8 | 3.78E-177 |

^a^ Baker YR, Hodgkinson JT, Florea BI, Alza E, Galloway W, Grimm L, Geddis SM, Overkleeft HS, Welch M, Spring DR. 2017. Identification of new quorum sensing autoinducer binding partners in *Pseudomonas aeruginosa* using photoaffinity probes*.* Chem Sci 8:7403-7411.
